# γδ T-cell interferon-γ production reflects metabolic improvement during SGLT2 inhibitor-based therapy in type 2 diabetes

**DOI:** 10.3389/fendo.2026.1922668

**Published:** 2026-09-11

**Authors:** Marija Troskot Cuculić, Dora Sergo, Inga Kavazović, Viktor Peršić, Felix M. Wensveen, Tamara Turk Wensveen

**Affiliations:** 1 Center for Diabetes, Endocrinology and Cardiometabolism, Special Hospital for Medical Rehabilitation of the Heart and Lung Diseases and Rheumatism Thalassotherapia Opatija, Opatija, Croatia; 2 Department of Histology and Embryology, Faculty of Medicine, University of Rijeka, Rijeka, Croatia; 3 Hospital for Medical Rehabilitation of Heart and Lung Diseases and Rheumatism Thalassotherapia-Opatija, Opatija, Croatia; 4 Department of Internal Medicine, Faculty of Medicine, University of Rijeka, Rijeka, Croatia; 5 Department of Endocrinology, Diabetes and Metabolic Diseases, Clinic of Internal Medicine, Clinical Hospital Center Rijeka, Rijeka, Croatia; 6 Department of Clinical Medicine, Faculty of Dental Medicine and Health, Josip Juraj Strossmayer University of Osijek, Osijek, Croatia

**Keywords:** diabetes mellitus type 2, gamma delta (gammadelta) T cells, GLP1 receptor agonist, IFN gamma, meta-inflammation, SGLT2 inhibitors, T cells, TNF

## Abstract

**Introduction:**

Chronic low-grade meta-inflammation contributes to insulin resistance and metabolic dysfunction in type 2 diabetes (T2D). γδ T cells have emerged as important mediators of metabolic inflammation, yet their responsiveness to metabolic improvement in humans remains poorly understood. We investigated whether successful antidiabetic therapy is associated with alterations in γδ T cell-mediated inflammation in patients with T2D.

**Methods:**

Twenty-five patients with poorly controlled T2D initiating SGLT2i therapy (n=15) or SGLT2i plus GLP-1RA (n=10) were prospectively followed for 12 months. Age- and sex-matched non-diabetic controls (n=30) were included at baseline. Peripheral blood γδ T cell phenotype and cytokine production were assessed by multiparametric flow cytometry. Metabolic, hepatic, and renal parameters were evaluated longitudinally.

**Results:**

At baseline, patients with T2D exhibited increased interferon-γ (IFN-γ) production by both Vδ1^+^ and Vδ2^+^ γδ T-cell subsets compared with controls, while subset frequencies remained unchanged. During follow-up, successful antidiabetic therapy was associated with a progressive reduction in IFN-γ production, reaching approximately 40% after 12 months (p<0.01). This reduction did not correlate with changes in HbA1c and was not enhanced by the addition of GLP-1 receptor agonist therapy. In contrast, lower IFN-γ production was associated with improved insulin sensitivity, reduced fat mass, and markers of improved metabolic health. The largest reductions were observed in individuals with mild hepatic or renal impairment and correlated with indices of organ involvement.

**Discussion:**

This real-world study shows that γδ T cell-mediated meta-inflammation decreases during successful antidiabetic therapy independently of glycemic control. IFN-γ production by γδ T cells is closely associated with insulin resistance, adiposity, and metabolic organ dysfunction, supporting its potential utility as a biomarker of metabolic tissue stress and therapeutic response in T2D.

## Graphical Abstract

**Figure:**
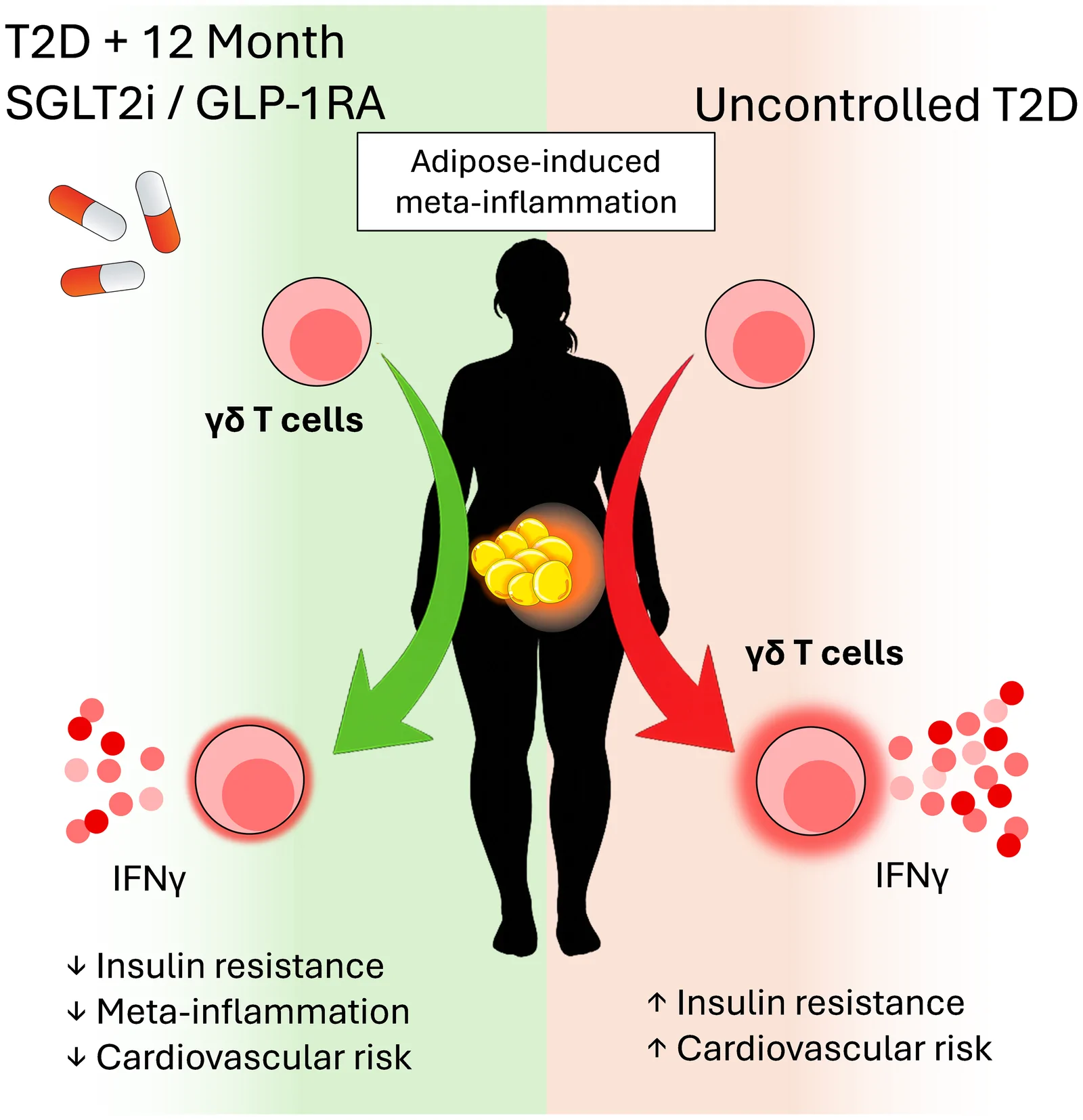

## Introduction

In recent years, the scope of antidiabetic treatment has shifted from a narrow focus on glucose control to a more comprehensive approach that includes additional health benefits such as weight management and the reduction of cardiorenal and cardiovascular risk (1, 2). The ADVANCE and UKPDS trials have shown that levels of glycated hemoglobin A_1c_ (HbA_1c_) positively correlate with the risk of microvascular disease, stroke, heart failure incidence and myocardial infarction (3, 4). In addition, a 1% reduction in HbA_1c_ was shown to translate to a 6% decrease in all-cause mortality among patients with type 2 diabetes mellitus (T2D), coupled with a notable 10% reduction in diabetes-related mortality (4). Modern antidiabetic drugs, such as sodium-glucose cotransporter-2 (SGLT2) inhibitors and glucagon-like peptide-1 receptor agonists (GLP-1RAs), have been shown to be particularly potent in reducing health risks in people with type 2 diabetes mellitus (T2D). Core clinical trials have demonstrated the efficacy of SGLT2i in reducing major adverse cardiovascular events, hospitalizations for heart failure and slowing the progression of chronic kidney disease. Similarly, GLP-1RAs enhance glucose-dependent insulin secretion and have shown significant cardiovascular benefits (5, 6).

Interestingly, the beneficial effects of modern antidiabetic drugs appear to depend on other factors than regulation of glycemia alone. Head-to-head comparative studies consistently show that SGLT2 inhibitors and GLP-1 receptor agonists are associated with lower rates of diabetic complications such as major adverse cardiovascular events (MACE) and heart failure hospitalization than older antidiabetic drugs such as DPP-4 inhibitors and sulfonylureas in routine clinical practice (7). These advantages persist after adjustment for baseline HbA_1c_ and other cardiometabolic risk factors, and for SGLT2 inhibitors, remain remarkably consistent across categories of glycemic control (8, 9). Together with cardiovascular and renal outcome trials demonstrating benefits in individuals without diabetes, such as the EMPEROR-preserved and SELECT trials (10, 11), these data support the notion that SGLT2 inhibitors and GLP-1 receptor agonists confer cardiovascular protection that is only partly explained by glucose lowering.

One possible mechanism via which SGLT2i and GLP-1RA mediate their beneficial effect is through control of inflammation. Metabolic disease is associated with chronic systemic inflammation, also referred to as meta-inflammation, due to tissue stress in organs such as liver and adipose tissue, which accumulate large amounts of nutrients (12). A large prospective cohort study including over 20.000 participants showed that elevated inflammatory markers such as high-sensitivity C-reactive protein (hsCRP) and interleukin (IL) 6 in circulation predict incident myocardial infarction, stroke, and cardiovascular mortality even in otherwise healthy individuals, outperforming several traditional risk factors (13). In patients with diabetes, higher CRP levels were an independent risk factor for the development of MACE (14). Of note, treatment of patients with SGLT2 inhibitors or GLP-1RAs resulted in a reduction of inflammatory markers (15, 16). Unfortunately, serum levels of molecules such as CRP and IL-6 are typically low, requiring assays of very high sensitivity and large patient groups to show differences. Moreover, these markers are associated with general inflammation and are not specific for the underlying pathophysiology of T2D. This precludes their use as a biomarker for disease status and therapy responsiveness. Thus, a more specific link between inflammation, diabetes and its treatment may therefore be beneficial for better diagnosis and efficiency of treatment.

Recent studies have identified γδ T cells as potentially important mediators of metabolic inflammation in diabetes, although their functional responses appear to be context dependent (17). In patients with T2D, circulating γδ T cells exhibit increased production of inflammatory cytokines, including IFNγ and TNF, which could be ameliorated through modulation of their metabolism (18, 19). In addition, hyperglycemia can promote γδ T-cell activation through interactions with dendritic cells, providing further evidence that γδ T-cell inflammatory responses are sensitive to the diabetic metabolic environment (20). Previously, our team found that cytokine production of specific immune cells including, interferon (IFN) γ production by blood γδ T cells was increased in patients with uncontrolled T2D compared to controls (19). IFNγ is a cytokine which has been causally linked with blood glucose and lipid regulation in animal models (21–23). Here, we investigated whether antidiabetic treatment with SGLT2 inhibitors alone, or in combination with GLP-1RA ameliorated immune cell hyper-reactivity. We observed that both for δ1 and δ2 γδ T cells IFNγ production significantly decreased for at least 12 months after initiation of treatment. Reduction of hyperreactivity positively correlated with insulin sensitivity and fat mass. Our findings provide a valuable new link between T2D and systemic inflammation with great potential as a future biomarker for therapy efficiency.

## Methods

### Subjects

Adult patients with T2D eligible for antidiabetic treatment modification, including SGLT2 inhibitors and GLP-1 receptor agonists as add-on therapy to metformin, were recruited from the Centre of Diabetes, Endocrinology and Cardiometabolism, the diabetes outpatient clinic at the Special Hospital Thalassotherapia Opatija, Croatia, between November 2020 and October 2023. The diagnosis of T2D was confirmed based on the latest American Diabetes Association (ADA) guidelines (24).

The presence of exclusion criteria was established through medical history, clinical examination, and initial laboratory evaluation, omitting patients with the following immune-modulating factors: diagnosis of type 1 diabetes mellitus or latent autoimmune diabetes in adults (LADA; indicated by decreased C-peptide levels or positive autoantibodies to glutamic acid decarboxylase or islet antigen 2), acute infection or recent vaccination (within the past 4 weeks), recurrent urinary tract infections, chronic inflammatory diseases, active malignant diseases, disorders affecting the hypothalamus-pituitary-adrenal axis, use of anti-inflammatory or immunosuppressive medications, advanced liver or kidney disease. Additionally, patients taking insulin, or more than two oral antidiabetic drugs were not included in the study. Participants who did not fulfill any exclusion criteria but did not meet the criteria for diabetes diagnosis (24) were included in the control group (**Supplementary Table 1**).

### Treatment assignment

**Figure 1:**
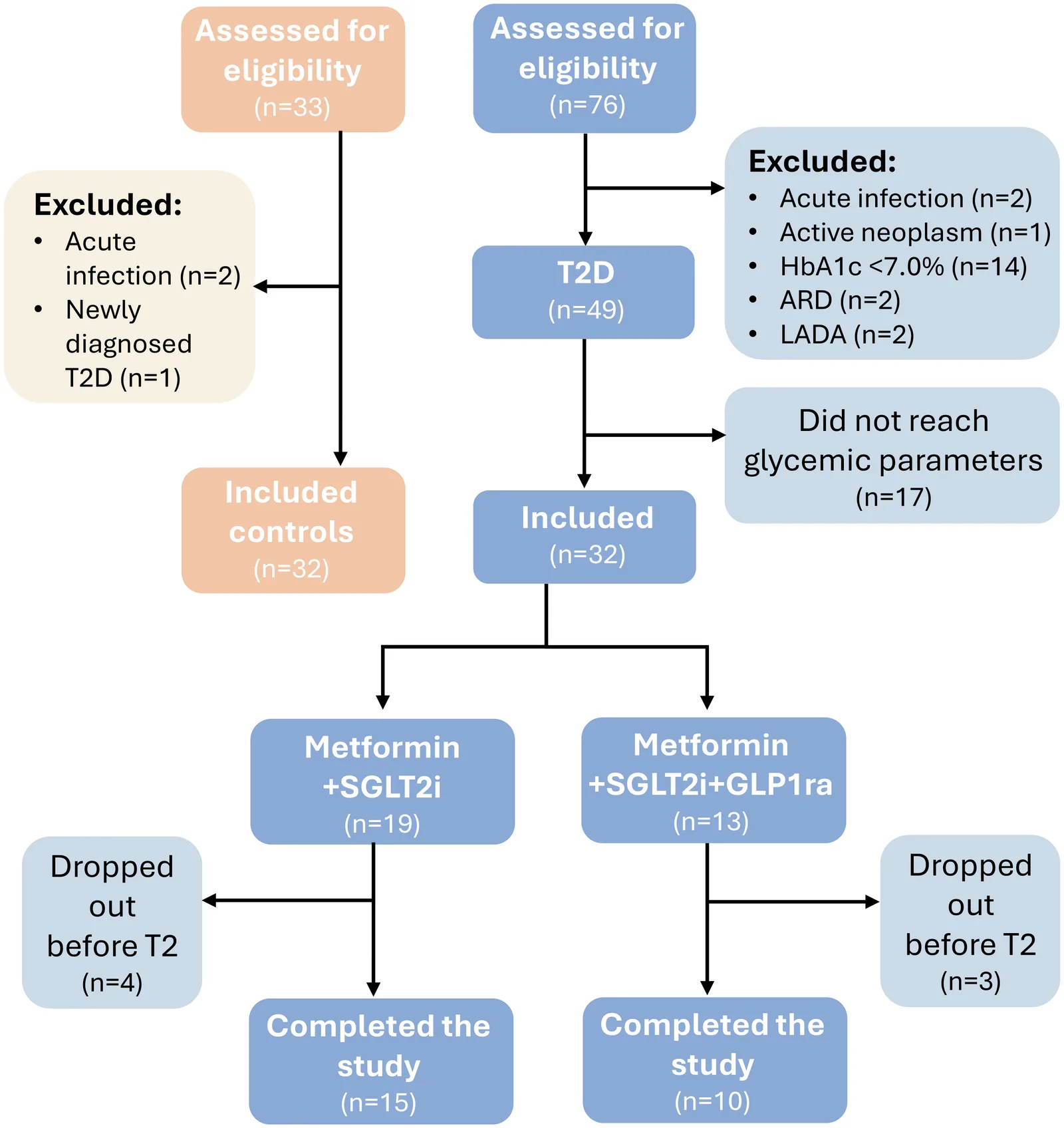
Study flowchart.

Patients with uncontrolled type 2 diabetes were treated with SGLT2 inhibitor (empagliflozin or dapagliflozin, administered once daily) either as monotherapy or in combination with GLP-1 receptor agonist semaglutide (administered once weekly by subcutaneous injection), in addition to a maximally tolerated dose of metformin. This was an observational study and assignment was done in accordance to standards of care guidelines (25). Monotherapy with GLP-1Ra was not included, as this is not a standard therapy for patients with T2D in Croatian clinical practice. Investigators were not blinded during the clinical phase of the study, but samples were blinded to investigators prior to laboratory analysis. If participants reached the target HbA_1c_ value of <7.0% after 3 months of modified antidiabetic treatment, blood samples were collected again at the 6-month mark. If the glycemic target was not achieved after 3 months, the treatment regimen or dosage was adjusted, and the follow-up period was extended to 9 months according to the study diagram (**Supplementary Figure 1**). If the glycemic target was not met 6 months after the antidiabetic treatment modification, if treatment was prematurely discontinued due to adverse effects, or if the patient was lost to follow-up, blood collection was not repeated at the 6-month point and the patient was excluded from the analysis. *A priori* differences between in- and excluded patients in basic metabolic and immunological parameters was confirmed to be absent (**Supplementary Table 2**). Primary outcome was the impact of successful anti-glycemic treatment on γδ T cel mediated meta-inflammation, which is why this protocol was followed. See also **Figure 1**.

### Ethical considerations

The ethics committee of the Institutional Review Board at Thalassotherapia Opatija approved this research under number 01-000-00-503/2-2020. We conducted the research in accordance and agreement with the International Conference on Harmonization Good Clinical Practice Guidelines and with the Declaration of Helsinki. All participants were over 18 years old and provided informed consent prior to inclusion in the study.

### Blood isolation

Peripheral venous blood was collected between 7 and 9 a.m. in ethylenediaminetetraacetic acid (EDTA) and serum gel activator tubes from individuals who had fasted for at least 10 hours. Antidiabetic medications were discontinued 48 hours before the blood collection. Hematological and biochemical analysis were conducted using standard methods. Serum was tested for autoantibodies to exclude patients with LADA. Peripheral blood mononuclear cells (PBMCs) were isolated from whole blood by density gradient centrifugation using Histopaque 1077 (Sigma-Aldrich, St. Louis, Missouri, United States) or Lymphoprep (ProteoGenix, Schiltigheim, France) within 6–8 hours of blood collection. Control experiments were performed, which demonstrated that differences in isolation medium did not impact the parameters measured here. After isolation, the cells were cryopreserved in fetal bovine serum (Corning, Inc., Corning, New York, United States) and 10% dimethyl sulfoxide (Sigma-Aldrich, St. Louis, Missouri, United States) until use.

### Flow cytometry

Cell surface and intracellular flow cytometric analyses was performed on thawed samples according to standard protocols. Briefly, cells were washed and stained with fluorochrome-conjugated antibodies for specific surface markers for 30 minutes at 4 °C in the dark. For intracellular staining, cells were first stained for surface markers for 30 minutes at 4 °C, followed by permeabilization and fixation using either the intracellular fixation and permeabilization set or the Foxp3 transcription factor fixation and permeabilization set (eBioscience, Inc., San Diego, California, United States). Intracellular molecules were then stained for 20 minutes at room temperature using anti-human antibodies. All antibodies used in the study are summarized in **Supplementary Table 3**. Per patient, samples from each timepoint were analyzed simultaneously as much as possible to prevent batch issues. Data were acquired using a BD FACSAriaIIu flow cytometer (BD, Franklin Lakes, New Jersey, United States) and analyzed with FlowJo software (FlowJo LLC, Ashland, Oregon, United States). The gating strategy followed the Guidelines for the use of flow cytometry and cell sorting in immunological studies (26). In brief, viable cells were identified by staining with a cell permeable viability dye and lymphocytes were gated based on FSC/SSC. Next, δ2 and δ1 γδ T cells were defined as CD3^+^TCR-δ1^+^δ2^-^ and CD3^+^TCR-δ1^+^δ2^-^ respectively (**Supplementary Figure 2**). The absolute reduction in IFNγ production was calculated by subtracting the percentage of cells producing this cytokine post treatment from the percentage prior to treatment. The relative reduction was calculated by dividing the difference on the fraction of cells producing IFNγ prior to treatment.

### *In vitro* stimulations

Upon thawing, isolated PBMCs were stained with a 0.4% trypan blue solution (ThermoFisher Scientific, Waltham, Massachusetts, United States), enumerated, and assessed for viability using CytoSMART (CytoSMART Technologies, Eindhoven, Netherlands). To measure cytokine production, cells were incubated at 37 °C with 5% CO2 in the presence of 20 ng/ml phorbol-12-myristate-13-acetate (PMA, Sigma-Aldrich, St. Louis, Missouri, United States) and 1µmol/L Ionomycin (PMA, Sigma-Aldrich, St. Louis, Missouri, United States) for 4 hours in the presence of Brefeldin A (eBioscience, San Diego, California, United States).

### Anthropometric measurements and body composition analysis

Anthropometric measurements included body weight, height, waist and hip circumference. Weight and height were measured using a high-precision digital column scale with a stadiometer (Seca, Hamburg, Germany). A standard cloth tape measure was used to record waist and hip circumference. Body composition was estimated using bioelectrical impedance analysis technology (InBody, Seoul, South Korea).

### Statistical analysis

Data are presented as paired individual values. Depending on the type of variables, normality of data distribution, and sample characteristics, statistical significance was determined by Fisher’s exact test, Chi-square test, Mann-Whitney test, Wilcoxon test, McNemar test, Fischer’s exact test and Kruskal-Wallis test followed by Dunn’s *post-hoc* test. Correlation analysis was used to determine the Spearman coefficient. A *p*-value <0.050 was considered statistically significant, with specific significance levels reported in the figure captions. To assess potential confounding by concomitant statin therapy and BMI, *post hoc* sensitivity analyses were performed using multiple linear regression. Because only two patients with T2D had a BMI <25 kg/m², BMI was analyzed as a continuous variable rather than by conventional BMI categories. To assess whether BMI confounded differences in baseline IFNγ production between patients with T2D and non-diabetic controls, baseline IFNγ production was modeled separately for Vδ1^+^ and Vδ2^+^ γδ T cells, with T2D status and BMI as covariates. To assess potential confounding of longitudinal treatment effects, IFNγ production at 12 months was modeled separately for Vδ1^+^ and Vδ2^+^ γδ T cells, including baseline IFNγ production, treatment group and either baseline BMI or initiation of statin therapy during follow-up as covariates. In addition, models using the absolute change in IFNγ production from baseline to 12 months as the dependent variable were performed as confirmatory analyses. Analyses were performed using GraphPad Prism (GraphPad Software, San Diego, California, United States) or R (v4.6.0) and R studio (v2026.05.1). Indices were calculated as follows; HOMA-IR: Fasting insulin (µU/mL; FPI) x Fasting Glucose (mmol/L; FPG)/22.5, HOMA-B: 20 x FPI/FPG-3.5; BMI: kg/m^2^ (height); Waist-hip ratio: waist circumference/hip circumference; Hepatic steatosis index: 8 x (ALT/AST) + BMI + 2 (+2 if female); Triglyceride-glucose index: ln x (Fasting triglycerides (mg/dL x FPG/2); NAFLD liver fat score: -2.89 + 1.18x1 + 0.45x2 + 0.15xFPI + 0.04xAST -0.94x(AST/ALT).

## Results

### Antidiabetic treatment ameliorates IFNγ production by γδ T cells

**Figure 2:**
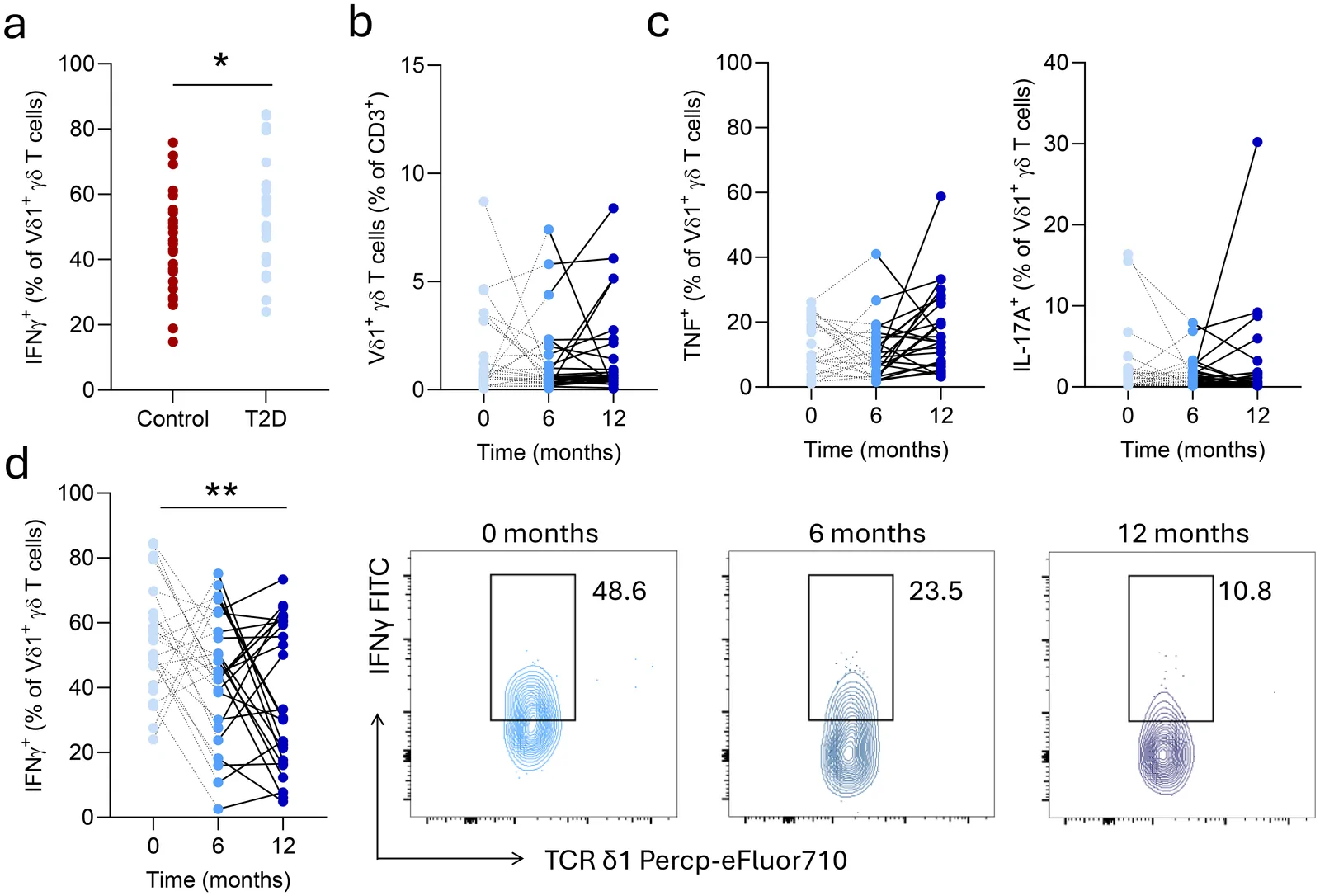
Antidiabetic treatment ameliorates IFNγ production by δ_1_^+^ γδ T cells. **(a)** Peripheral blood mononuclear cells from patients with type 2 diabetes mellitus (n=25) and matched controls (n=30) were stimulated and interferon (IFN) γ production by δ_1_^+^ γδ T cells was determined using intracellular flow cytometry at baseline. **(b)** δ_1_^+^ γδ T cells as a frequency of total CD3^+^ T cells after antidiabetic treatment (n=25). **(c)** Tumor necrosis factor (TNF) and interleukin **(IL)** 17A production by peripheral blood δ_1_^+^ γδ T cells upon *in vitro* restimulation after indicated times of antidiabetic treatment (n=25). **(d)** IFNγ production by peripheral blood δ_1_^+^ γδ T cells upon *in vitro* restimulation after indicated times of antidiabetic treatment (n=25). Shown are representative fluorescence activated cell sorting plots of CD3^+^δ_1_^+^ cells from the same patient over time. An individual point represents one participant. Indicated are mean values ± standard error of mean and statistical significances at *p < 0.050 and **<0.010 by **(a)** Mann-Whitney and **(d)** one-way ANOVA with Tukey’s multiple comparisons post testing.

Previously, we have shown that T2D does not impact the frequency of γδ T cells in the blood of patients, but causes an increase in the production of cytokines (19). Indeed, prior to treatment, we observed that T-cell receptor (TCR) δ1^+^ γδ T cells isolated from the blood of patients with T2D expressed higher levels of IFNγ then cells of matched controls upon *in vitro* restimulation with PMA and ionomycin (**Figure 2a**; **Supplementary Table 4**). Because BMI differed between patients with T2D and controls, we performed a sensitivity analysis to assess whether BMI contributed to the observed difference in IFNγ production. After adjustment for BMI, T2D remained independently associated with higher IFNγ production by Vδ1^+^ γδ T cells, whereas BMI itself was not independently associated with IFNγ production (**Supplementary Table 5a**).

Patients were segregated into two groups, one treated with metformin and SGLT2 inhibitors alone (SGLT2i) or in combination with GLP1-R agonists (Chart 1). As expected, apart from the improvement of glycemic parameters, which were used as a selection criterium for effective treatment, we observed significant improvement of the serum lipid profile of patients. In addition, we observed improvement in liver parameters, including reduced aspartate aminotransferase (AST), alanine aminotransferase (ALT) and hepatic steatosis index, whereas renal function was not significantly impacted (**Table 1**).

**Table 1:** Patient characteristics before the study and after 6 and 12 months of treatment. .

Variables	Baseline (*n* = 25)	6 months (*n* = 25)	12 months (*n* = 25)	0 vs. 6 months p value	0 vs. 12 months p value
Age, years	60 ± 8 (44-74)				
Female sex, n (%)	10 (40)				
Glycated hemoglobin A_1c_, %	8.3 ± 1.1	6.5 ± 0.4	6.6 ± 0.4	<0.001	<0.001
Fasting plasma glucose, mmol/l	9.3 ± 2.1	6.9 ± 1.1	6.9 ± 0.8	<0.001	<0.001
HOMA-insulin resistance	7.9 ± 4.5	4.6 ± 3.0	4.5 ± 3.3	<0.001	<0.001
HOMA-beta cell function	7.5 ± 4.1	8.9 ± 0.4	8.1 ± 4.9	0.116	0.465
Quantitative insulin sensitivity check index	0.29 ± 0.02	0.32 ± 0.03	0.32 ± 0.03	<0.001	<0.001
Body mass index, kg/m^2^	30.7 ± 4.1	28.5 ± 3.4	28.4 ± 3.7	<0.001	<0.001
Bodyweight	89.3 ± 16.8	84.3 ± 14.8	85.4 ± 15.1	0.006	0.067
Body fat, %	29.8 ± 8.7	28.4 ± 9.2	29.5 ± 8.4	0.208	0.733
Skeletal muscle index, kg/m^2^	8.8 ± 1.3	8.3 ± 1.1	8.2 ± 1.1	0.001	<0.001
Waist-hip ratio	0.97 ± 0.07	0.95 ± 0.06	0.97 ± 0.05	0.071	0.552
Leukocyte count, ×10^9^ per liter	7.7 ± 2.2	8.1 ± 2.1	8.3 ± 2.1	0.167	0.056
C reactive protein, mg/l	2.9 ± 2.4	2.2 ± 2.7	1.9 ± 1.9	0.128	0.114
Triglycerides, mmol/l	1.8 ± 1.2	1.6 ± 1.0	1.6 ± 0.9	0.491	0.473
HDL cholesterol, mmol/l	1.2 ± 0.4	1.3 ± 0.3	1.4 ± 0.3	0.010	<0.001
LDL cholesterol, mmol/l	2.8 ± 1.0	2.2 ± 1.0	2.2 ± 1.1	0.005	0.003
Total cholesterol, mmol/l	4.6 ± 1.3	4.2 ± 1.2	4.0 ± 1.3	0.042	0.024
Statin use, *n* (%)	13 (52)	23(92)	23 (92)	0.002	0.002
Aspartate aminotransferase, U/l	29.2 ± 13.5	25.5 ± 11.4	26.3 ± 10.5	0.149	0.250
Alanine aminotransferase, U/l	38.3 ± 22.4	28.0 ± 10.4	27.8 ± 11.3	0.009	0.013
Hepatic steatosis index	44.1 ± 6.1	40.4 ± 4.8	39.9 ± 4.8	<0.001	<0.001
Triglyceride-glucose index	9.3 ± 0.6	8.9 ± 0.6	8.9 ± 0.6	<0.001	0.001
NAFLD-liver fat score	2.5 ± 1.8	1.5 ± 1.5	1.4 ± 1.6	0.004	0.001
Urea, mmol/l	5.9 ± 1.3	6.2 ± 0.4	6.5 ± 1.9	0.391	0.096
Creatinine, µmol/l	76.1 ± 13.4	72.3 ± 16.8	75.0 ± 18.2	0.026	0.595
eGFR, ml/min/1.73m^2^	83.4 ± 15.7	88.7 ± 16.2	87.0 ± 17.9	0.006	0.090

Next, we investigated whether antidiabetic treatment had an impact on meta-inflammation mediated by γδ T cells. PBMCs were isolated from patients at time of initial visit, after 6 months of treatment and after 12 months of treatment. Cells from each timepoint were stimulated simultaneously and their phenotype and function were analyzed. In the blood, most γδ T cells express either the TCR δ1 or δ2 chain, with the latter type typically being prevalent approximately five times more. δ1^+^ T cells are thought to predominantly produce cytokines, such as IFNγ, tumor necrosis factor (TNF) and IL-17A, whereas δ2^+^ cells are considered to have higher cytotoxic potential and express more Granzyme B. We first analyzed δ1^+^ γδ T cells. Antidiabetic treatment did not impact the frequency of these cells as a fraction of total CD3^+^ T cells (**Figure 2b**). Expression of the costimulatory molecules CD27 and CD28 has been associated with the ability to produce specific cytokines such as IL-17A, both in humans and mice (27). However, no changes were observed upon antidiabetic treatment (**Supplementary Figure 3a**). CD16, a protein associated with antibody-dependent cytotoxicity (28) was also present in equal amount on the surface of δ1^+^ γδ T cells at all time points after initiation of treatment (**Supplementary Figure 3b**). Finally, expression of CD45RA, as a marker of terminal effector differentiation, was likewise not affected by any of the time points (**Supplementary Figure 3c**). Thus, antidiabetic treatment did not appear to impact the phenotype of δ1^+^ γδ T cells.

To determine whether antidiabetic treatment impacts immune cell responsiveness, cells were restimulated *in vitro* using PMA and ionomycin and cytokine production was assessed by intracellular flow cytometry. Antidiabetic treatment did not impact the ability of δ1^+^ γδ T cells to produce either TNF or IL-17A (**Figure 2c**). In contrast, we observed a strong impact on the production of IFNγ. At six months of treatment, we observed that on average there were 11% (± 26.3) less δ1^+^ γδ T cells that produced IFNγ (absolute reduction). This translated to relative reduction (i.e. compared to the fraction of δ1^+^ γδ T cells producing IFNγ prior to treatment) of 20% (± 48.0). After 12 months, the absolute reduction was 17.2% (± 17.1%) whereas the relative reduction was 39.2% (± 31.2), which reached statistical significance at this point (**Figure 2d**; **Supplementary Figure 3d**).

**Figure 3:**
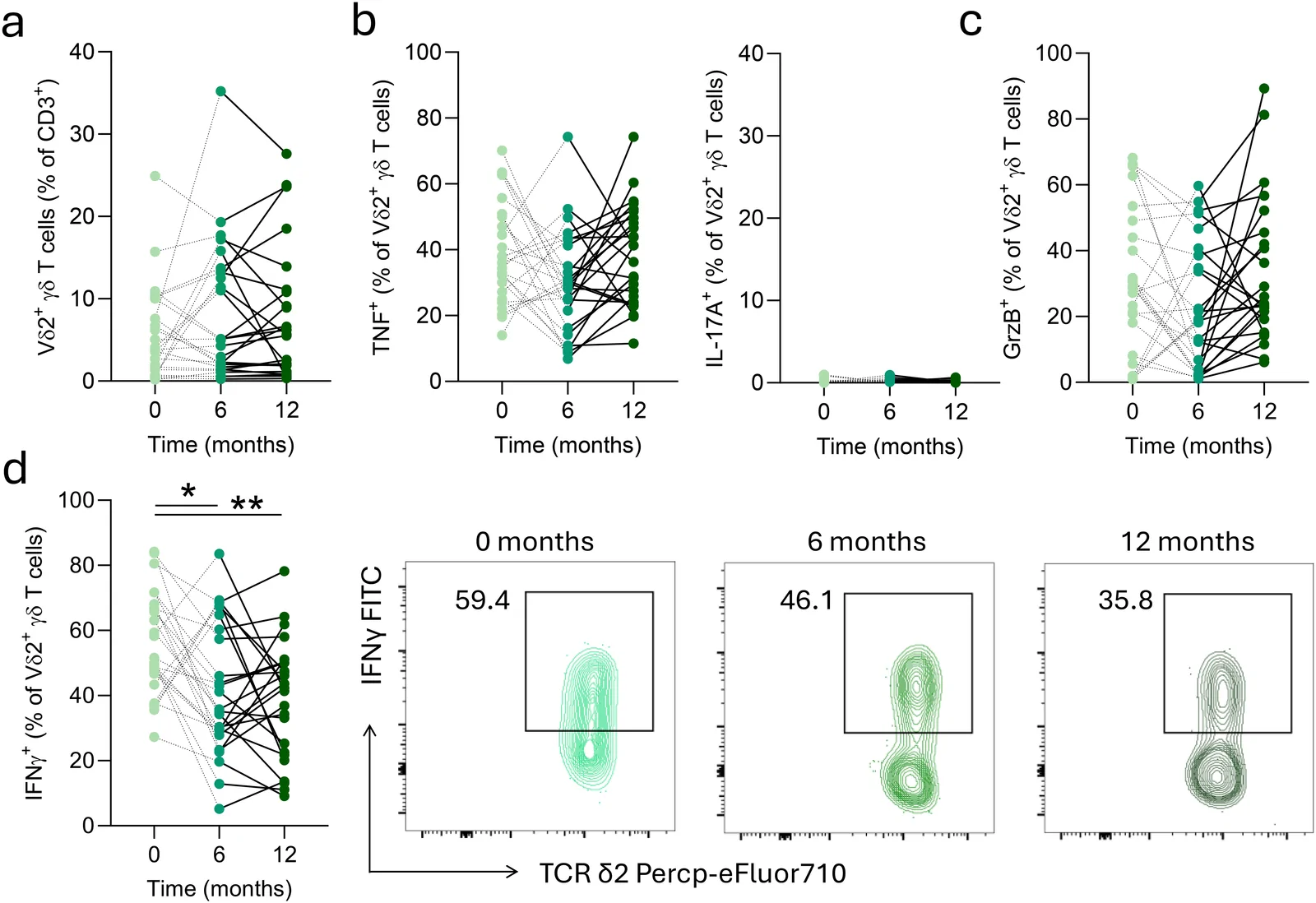
Antidiabetic treatment ameliorates IFNγ production by δ_2_^+^ γδ T cells. **(a)** δ_2_^+^ γδ T cells as a frequency of total CD3^+^ T cells after antidiabetic treatment (n=25). **(b)** Tumor necrosis factor (TNF) and interleukin (IL) 17A and **(c)** Granzyme B production by peripheral blood δ_2_^+^ γδ T cells upon *in vitro* restimulation after indicated times of antidiabetic treatment (n=25). **(d)** Interferon (IFN) γ production by peripheral blood δ_2_^+^ γδ T cells upon *in vitro* restimulation after indicated times of antidiabetic treatment (n=25). Shown are representative fluorescence activated cell sorting plots of CD3^+^δ_2_^+^ cells from the same patient over time. An individual point represents one participant. Indicated are mean values ± standard error of mean and statistical significances at *p < 0.050 and **<0.010 by one-way ANOVA with Tukey’s multiple comparisons post testing.

Subsequently, we investigated how antidiabetic treatment impacts δ2^+^ γδ T cells. Neither at 6, nor at 12 months of treatment did we observe changes in the frequency of these cells as a fraction of CD3^+^ T cells (**Figure 3a**). Phenotypic analysis showed a minor shift of a more active CD28^+^CD27^+^ to a more anergic CD28^-^CD27^-^ phenotype (**Supplementary Figure 3e**). This corresponded with a minor increase in expression of CD45RA at 12 months of treatment. Expression of CD16 was not affected on these cells at any time point (**Supplementary Figures 3f, g**).

Similar to δ1^+^ γδ T cells, we did not observe changes in the ability of δ2^+^ γδ T cells to produce TNF. As expected, these cells secrete very little IL-17A, which was not impacted by antidiabetic treatment. Instead, these cells produce Granzyme B, which was also not affected. Again, we observed a strong impact of antidiabetic treatment on production of IFNγ. Already after 6 months we observed a significant reduction in the production of this cytokine (**Figure 3d**; **Supplementary Figure 3h**), with an absolute reduction of 14.1% (± 25.1) and a relative reduction 25.1% (± 44.8). This reduction was slightly increased at 12 months, with an absolute reduction of 17.4% (± 16.5) and a relative reduction of 41.5% (± 29.5). Neither the cumulative amount of δ1^+^ and δ2^+^ γδ T as a percentage of CD3ϵ^+^ cells, nor the ratio between δ1^+^ and δ2^+^ γδ T cells was impacted by anti-diabetic treatment (**Supplementary Figures 3i, j**).

Thus, antidiabetic treatment does not impact the frequency of γδ T cells and only has a mild impact on their phenotype. In contrast, antidiabetic treatment ameliorates the hyperresponsiveness of γδ T cells, with a specific impact on IFNγ production.

### GLP1R agonists add-on therapy does not show a detectable increase in anti-inflammatory effects

Both SGLT2 inhibitors and GLP-1R agonists have a broad, beneficial impact on metabolic parameters in patients with T2D. To investigate whether they have a compound effect, we segregated these groups based on whether they received SGLT2 inhibitors only or in combination with GLP-1R agonists. As expected, add-on GLP-1R agonists induced greater metabolic changes than SGLT2 treatment alone. SGLT2 inhibition alone or in combination with GLP-1RA inhibition resulted in an average reduction of fasting plasma glucose of 1.52 (± 1.08) and 3.58 (± 3.25) mmol/L, whereas BMI was reduced by 1.76 (± 1.51) and 3.03 (± 2.88) kg/m^2^ respectively. Next, we analyzed cytokine production by γδ T cells in these groups. Segregation in subgroups did not reveal an independent effect of antidiabetic drugs on the production of TNF or IL-17A by δ1^+^ γδ T cells (**Supplementary Figures 4a, b**). Neither did it uncover and effect on production of TNF and Granzyme B by δ2^+^ γδ T cells (**Supplementary Figures 4c, d**).

**Figure 4:**
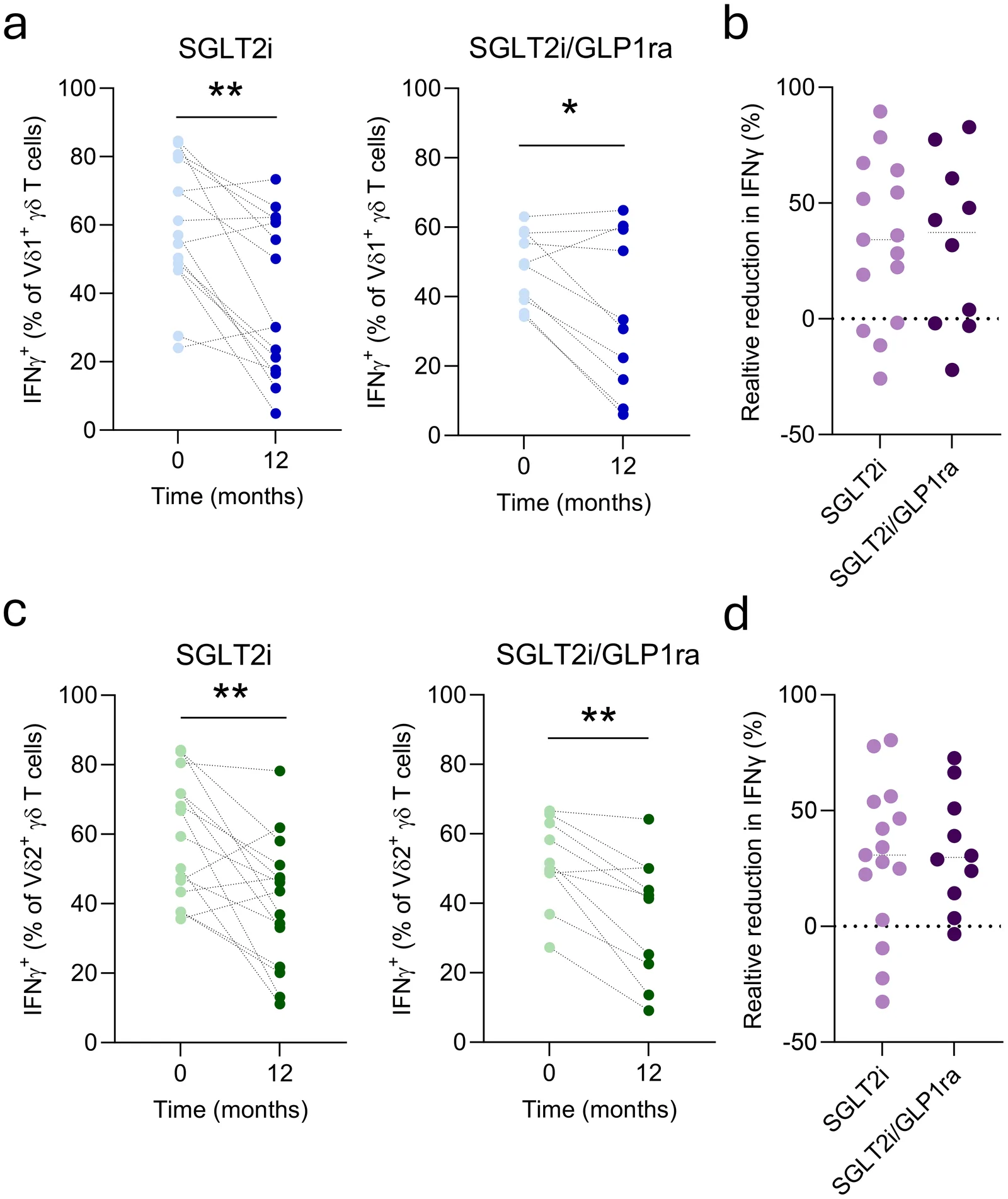
Combined treatment does not further ameliorate meta-inflammation. Groups were segregated based on treatment regime and interferon (IFN) γ production was assessed using intracellular flow cytometry. **(a)** IFNγ production by δ_1_^+^ γδ T cells at 0 and 12 months (n=15 and 10) **(b)** Relative reduction in IFNγ production by δ_1_^+^ γδ T cells (n=15 and 10). **(c)** IFNγ production by δ_2_^+^ γδ T cells at 0 and 12 months (n=15 and 10). **(d)** Relative reduction in IFNγ production by δ_2_^+^ γδ T cells (n=15 and 10). An individual point represents one participant. Indicated are mean values ± standard error of mean and statistical significances at *p < 0.050 and **<0.010 by paired t test.

We then focused on IFNγ. Patients treated with SGLT2 inhibitors on average showed a 20% (± 18.4) reduction in the frequency of δ1^+^ γδ T cells producing IFNγ upon *in vitro* restimulation (**Figure 4a**). Add-on treatment with incretin mimetics did not cause a further reduction in the secretion of IFNγ (12.9% ± 13.9). Indeed, the relative reduction in IFNγ production was not significantly different between treatment groups (33.4% ± 33.3 vs. 32.0% ± 34.5, **Figure 4b**).

Comparable observations were made for δ2^+^ γδ T cells. The group treated with SGLT2 inhibitors only showed an absolute reduction in IFNγ production of 18.7% (± 19.4) versus 15.5% (± 10.5) in the group receiving add-on GLP1R agonists (**Figure 4c**). This resulted in a relative reduction of 29.0% (± 32.2)and 32.7% (± 23.8) in patients treated with SGLT2i alone or in combination with GLP1R agonists, which was not significantly different between these two groups (**Figure 4d**).

Because statin therapy was initiated in a subset of participants during follow-up and BMI may influence γδ T-cell function, we performed sensitivity analyses to assess their potential contribution to the observed changes in IFNγ production. In baseline-adjusted multivariable models, neither statin initiation nor baseline BMI was independently associated with IFNγ production at 12 months in either γδ T cell subpopulation. Importantly, adjustment for either factor did not alter the absence of an additional effect of GLP-1RA therapy on IFNγ production. Comparable results regarding treatment group were obtained when absolute changes in IFNγ production were analyzed. In this secondary model, statin initiation was associated with a modest reduction in IFNγ production by Vδ2^+^, but not Vδ1^+^, γδ T cells, whereas baseline BMI was not associated with changes in IFNγ production in either subset (**Supplementary Tables 5b,c**).

In summary, treatment of patients with SGLT2 inhibitors is associated with a significant reduction of γδ T cell hyper-responsiveness. We could not demonstrate further amelioration by GLP1 receptor agonist add-on therapy.

### IFNγ levels correlate with changes in HOMA-IR and fat mass

**Figure 5:**
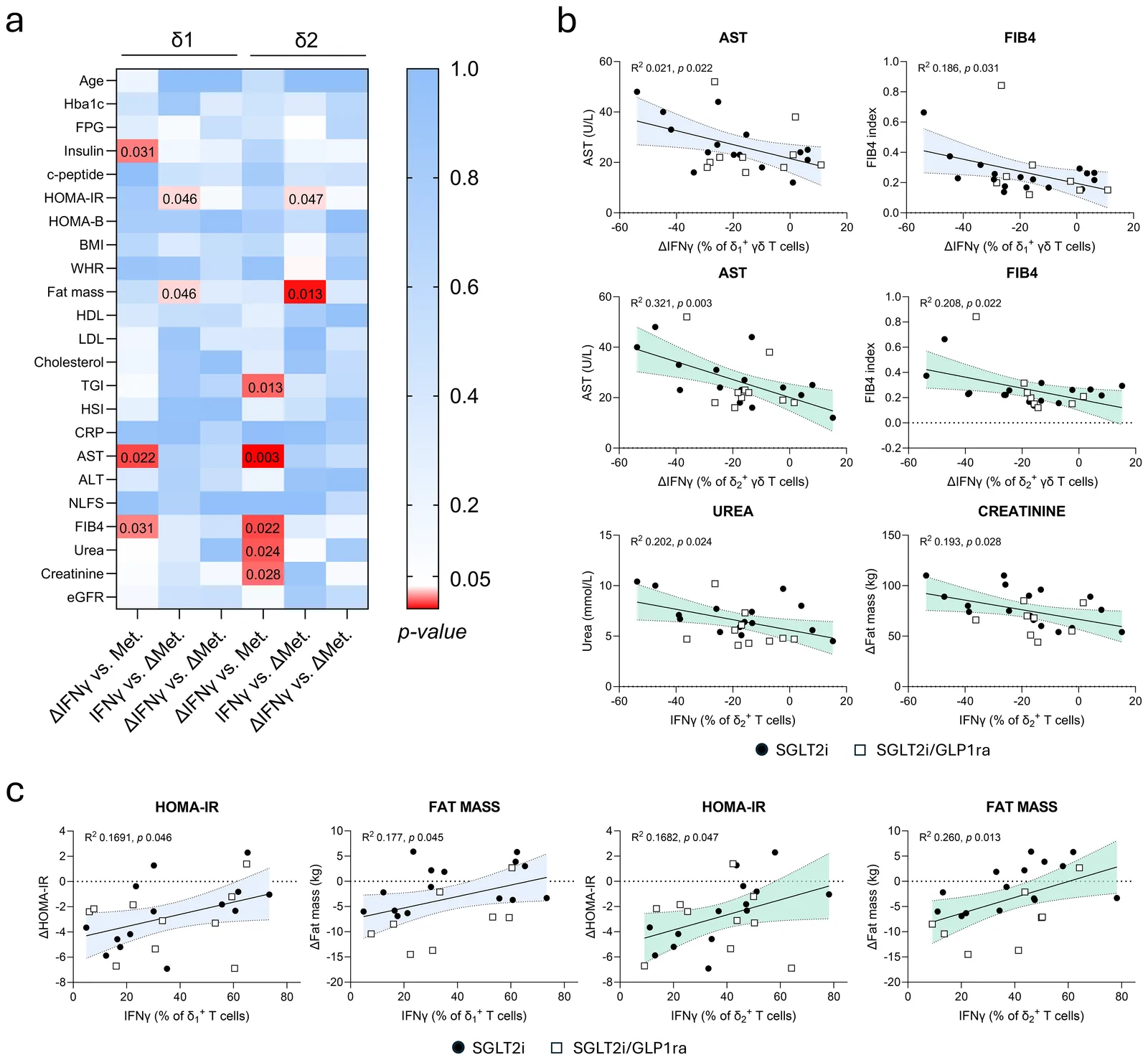
Reduced meta-inflammation correlates with reduced HOMA-IR and fat mass 12 months after treatment. *Post hoc* analysis of correlation between absolute levels of interferon (IFN) γ production and metabolic parameters, or relative change of these values (Δ) after 12 months of treatment. **(a)** Heat map of p values. Statistically significant changes are provided as numerical values (n=25). **(b)** Regression analysis correlating changes in IFNγ production with metabolic parameters (n=25). **(c)** Regression analysis correlating IFNγ production with changes in metabolic parameters. Regression analyses with statistically significant *p* value at <0.050 represented as mean and 95% confidence area of the best-fit line (n=25). ALT, alanine aminotransferase; AST, aspartate transaminase; BMI, body mass index; CRP, C-reactive protein; eGFR, estimated glomerular filtration rate FIB4, fibrosis-4 index; FPG, fasting plasma glucose; HbA_1c_, glycated hemoglobin A_1c_; HDL, high-density lipoprotein; HOMA-B, homeostatic model assessment beta cell function; HOMA-IR, homeostatic model assessment insulin resistance; HSI, hepatic steatosis index; LDL, low-density lipoprotein; NLFS, non-alcoholic fatty liver disease liver fat score; TyG triglyceride index; WHR, waist-hip ratio.

We subsequently wanted to elucidate which metabolic parameters may be associated with changes in the inflammatory status of γδ T cells. We therefore performed a *post hoc* analysis to identify correlations between IFNγ production and physiological parameters associated with metabolic syndrome. Changes in IFNγ did not correlate with relative alterations in any metabolic parameter investigated (**Supplementary Figure 5a**). We therefore first wanted to explore in which patient subgroup alterations of IFNγ were most prominent. We correlated changes in IFNγ with absolute levels of metabolic parameters. Advanced liver and kidney pathology were exclusion criteria for this study. Nevertheless, we observed that the patients which showed the greatest reduction in IFNγ production were those with the highest liver damage indices, such as increased serum levels of AST and a higher FIB4 fibrosis index. This was the case both for δ1^+^ and δ2^+^ γδ T cells (**Figures 5a, b**). Importantly, for δ2^+^ γδ T cells, changes in IFNγ also correlated with increased urea and creatinine levels, which may be indicators of mild chronic kidney disease. Notably, changes in IFNγ expression also correlated with liver and kidney pathology prior to treatment, both for δ1^+^ and δ2^+^ γδ T cells (**Supplementary Figures 5b–d**), as indicated by a positive correlation with FIB4 and serum urea levels.

To gain insight in the potential impact of IFNγ produced by γδ T cells on metabolic syndrome, we correlated absolute expression levels of this cytokine with changes in the values of metabolic parameters. We observed that IFNγ levels positively correlated with changes in the HOMA-IR index, as well as with changes in fat mass. We observed this both for δ1^+^ and δ2^+^ γδ T cells (**Figure 5c**).

Thus, changes in IFNγ production by γδ T cells were most prominent in patients with mild pre-existing liver and kidney damage. Moreover, the level of IFNγ expression by these cells corresponded with adipose tissue mass, as well as insulin resistance, but not with other parameters of metabolic syndrome.

## Discussion

Previous studies have examined the effects of SGLT2i and GLP-1RA mediated antidiabetic therapies on systemic markers of inflammation (29, 30). However, their impact on immune cell populations directly implicated in metabolic pathology has remained largely unexplored. Here, we demonstrate that treatment with SGLT2 inhibitors alone, or in combination with GLP-1 receptor agonists is associated with a marked reduction in IFN-γ production by γδ T cells. Importantly, this reduction in γδ T cell–mediated meta-inflammation was closely associated with improved insulin sensitivity and a decrease in fat mass, identifying IFN-γ production by these cells as a potential biomarker of therapeutic responsiveness in type 2 diabetes.

Both SGLT2 inhibitors and GLP-1 receptor agonists are known to reduce cardiovascular risk, even in individuals without established type 2 diabetes, and these benefits emerge rapidly after treatment initiation (10, 31). Chronic inflammation is a recognized driver of cardiovascular disease. However, antidiabetic therapies exert only modest effects on conventional inflammatory markers such as CRP and IL-6 (29, 30). This likely reflects the fact that within the immunological response, these proteins are primarily associated with acute inflammatory responses, particularly following bacterial infection, and are only weakly induced by the chronic, low-grade inflammatory processes characteristic of metabolic disease (32, 33). Accordingly, detection of treatment-induced changes in these markers often requires high-sensitivity assays (34). In contrast, we and others have previously shown in experimental models that metabolic stress in organs such as liver and adipose tissue is sensed by innate-like lymphocytes, including natural killer cells and γδ T cells (35, 36). Activation of these relatively small immune-cell populations can promote and amplify subsequent inflammatory responses involving more abundant effector populations. Indeed, experimental disruption of stress sensing by innate-like lymphocytes markedly reduces tissue inflammation and metabolic pathology (32, 33). Thus, the biological relevance of γδ T cells may derive from their role as sensors and regulators of metabolic tissue stress rather than from their quantitative contribution to systemic cytokines. IFN-γ produced by these cells promotes local inflammation and directly contributes to insulin resistance (22). Consistent with this, we observed up to a 40% reduction in IFN-γ production by γδ T cells following treatment, which correlated with reduced fat mass and improved insulin sensitivity. The absence of a comparable effect on tumor necrosis factor production may reflect the predominant role of macrophages, rather than γδ T cells, as the main source of this cytokine in metabolic disease (37). The reduction in IFN-γ production observed here may not be restricted to γδ T cells, as metabolic improvement is likely to affect multiple immune-cell populations. Whether comparable functional changes occur in conventional CD4^+^ and CD8^+^ T cells, and how these relate to metabolic improvement, requires a systematic analysis beyond the scope of the present study. Together, these findings support IFN-γ production by γδ T cells as a sensitive cellular readout of metabolic tissue stress and meta-inflammatory improvement, irrespective of their quantitative contribution to systemic IFN-γ production.

Recent studies further support a role for γδ T cells in metabolic inflammation. Li et al. reported increased inflammatory cytokine production by circulating γδ T cells in patients with T2D, including increased IFN-γ production by Vδ2 T cells (18). These findings complement our longitudinal observations by demonstrating that γδ T-cell inflammatory activity is metabolically regulated, whereas our data show that this phenotype is reduced during successful antidiabetic treatment in patients with T2D. Other γδ T-cell responses may involve distinct mechanisms and effector functions. For example, hyperglycemia promotes a CD137L-CD137 interaction between dendritic cells and γδ T cells, resulting in increased γδ T-cell proliferation and IL-17A production in diabetic periodontitis (20). We observed no change in IL-17A production, while IFN-γ was markedly reduced. This difference may reflect the distinct disease settings and the analysis of tissue-associated versus circulating γδ T cells. Collectively, these studies support the emerging concept that γδ T-cell responses in diabetes depend on cellular subset, tissue localization and disease context (17).

Both SGLT2 inhibitors and GLP1 receptor agonists have previously been reported to individually ameliorate meta-inflammation in patients with type 2 diabetes (15, 29). Surprisingly, combination therapy was not associated with greater improvements in systemic inflammatory markers compared with monotherapy, even though to date this has not been a primary outcome of any comparative study (38). Accordingly, we did not observe a further reduction in IFN-γ production in patients receiving combined SGLT2 inhibitor and GLP-1 receptor agonist therapy compared with those treated with SGLT2 inhibition alone. One possible explanation is that γδ T cell-mediated meta-inflammation primarily reflects the absolute metabolic state of affected tissues, particularly adipose tissue, rather than relative changes in metabolic parameters (39). Although patients receiving combination therapy showed larger relative improvements with regards to metabolic parameters, they also presented with more severe disease at baseline. Following treatment, absolute measures such as glycemia and body mass index were comparable between groups (**Supplementary Table 6**).

It should be noted that significantly more patients used statins at the end of the study that at time of inclusion. However, in a sensitivity analysis adjusting for baseline IFNγ production, treatment group and initiation of statin therapy, statin initiation was not independently associated with IFNγ production at 12 months, while adjustment for statin initiation did not alter the absence of an additional effect of GLP-1RA therapy. Thus, a major role of statins in regulation of γδ T cell mediated meta-inflammation appears unlikely.

The mechanism underlying the reduction in γδ T-cell IFN-γ production remains to be determined. A direct effect of the antidiabetic drugs used in this study on γδ T cells cannot be excluded, but appears unlikely, as these cells do not express SGLT2 or the GLP-1 receptor (40). Instead, we propose that reduced cytokine production reflects alleviation of metabolic tissue stress. Using dietary models of obesity, we have previously shown that excessive lipid accumulation in adipose tissue and liver induces the expression of cellular stress ligands that activate innate-like lymphocytes, including NK cells and γδ T cells (23, 35, 36). Cytokines produced by these activated lymphocytes can, in turn, promote insulin resistance and metabolic dysfunction. As SGLT2 inhibitors and GLP-1 receptor agonists reduce lipid accumulation in metabolically stressed tissues, this may decrease the signals driving innate-like lymphocyte activation and thereby reduce γδ T-cell IFN-γ production. Although this model is consistent with our present findings, tissue inflammation was not directly assessed and dedicated mechanistic studies will be required to establish this relationship in humans. Nevertheless, IFNγ production by γδ T cells appears to be strongly and consistently downregulated in T2D patients with good therapy responsiveness, thus making it a potential biomarker for assessment of meta-inflammation in these patients.

### Limitations of the study

Limitations of this study include its specific focus on γδ T cells and a relatively restricted set of cytokines. Whereas γδ T cells have been associated with the underlying pathophysiology of T2D, other immune cells are also involved and have not been studied here as they were outside of the scope of this study. Moreover, γδ T cells are known to produce other cytokines than IFNγ, TNF and IL-17A, which should be considered in future studies. Also, our study has a relatively small number of participants, which may impact our ability to draw conclusions with regards to the general population of people with T2D and increases risk of a type II error. The study may also be too underpowered to demonstrate subtle effects on meta-inflammation beyond IFNγ. In particular, the subgroup comparison between SGLT2i alone and SGLT2i plus GLP-1RA was too underpowered to exclude small or moderate differences between treatments. The absence of a significant additional effect of GLP-1RA should therefore be interpreted as a failure to detect such an effect rather than evidence of therapeutic equivalence. Apart from being treated with SGLT2i and GLP1RA, our patients received metformin and other drugs for treatment of their comorbidities. Although sensitivity analyses did not indicate that initiation of statin therapy accounted for the main treatment-group findings, the limited cohort size precluded comprehensive multivariable adjustment for all concomitant therapies. An additional impact of statins as well as other medications on meta-inflammation can therefore not be excluded. Similarly, our sensitivity analysis did not demonstrate an association between BMI and IFNγ production by γδ T cells, but the small cohort size did not allow us to stratify patients based on BMI. Our study did not distinguish between people of different genders and race, which should be take into account when considering the translatability to the general population. Finally, this real-world study excluded patients who required treatment intensification with insulin after failing to achieve predefined glycemic targets, as insulin treatment was a predefined exclusion criterion because of its potential effects on immune-cell function. Patients included in and excluded from the longitudinal analysis did not significantly differ in the examined metabolic parameters or in baseline IFN-γ production by Vδ1^+^ and Vδ2^+^ γδ T cells. Nevertheless, our study cannot determine whether γδ T-cell hyper-responsiveness also improves in patients who fail to achieve glycemic targets, and our findings should therefore be interpreted in the context of patients responding successfully to SGLT2i- and GLP-1RA-based therapy.

## Conclusions

Our findings show that treatment of patients with type 2 diabetes with SGLT2 inhibitors alone or in combination with GLP1 receptor agonists ameliorates meta-inflammation mediated by γδ T cells. This effect correlated with improved insulin sensitivity and a reduction in fat mass. Our findings may explain why these drugs have an anti-inflammatory, cardioprotective effect, despite being primarily used to lower blood glucose levels.

## Data Availability

The raw data supporting the conclusions of this article will be made available by the authors, without undue reservation.

## Supplementary material

Supplementary Table 1 Inclusion and exclusion criteria of the study. COPD, Chronic obstructive pulmonary disease; HbA_1c_, Glycated hemoglobin A_1c_; HPA, Hypothalamus-pituitary-adrenal axis; IBD, Inflammatory bowel disease; LSM, Liver stiffness measure; RA, Rheumatoid arthritis.

Supplementary Table 2 Characteristics of in- and excluded patients at baseline. Baseline demographic, metabolic and immunological characteristics of patients included in the final longitudinal analysis and patients subsequently excluded from longitudinal follow-up. No statistically significant differences were observed between groups for any of the examined parameters. IFN-γ production represents the percentage of Vδ1+ or Vδ2+ γδ T cells producing IFN-γ following *in vitro* stimulation.

Supplementary Table 3 List of antibodies used in experiments.

Supplementary Table 4 Characteristics of patients and controls at baseline. eGFR, estimated glomerular filtration rate; HDL, high-density lipoprotein; HOMA, homeostatic model assessment; LDL, Low-density lipoprotein.

Supplementary Table 5 Sensitivity analyses of potential confounding by statin initiation and BMI. Multiple linear regression analyses were performed to assess potential confounding of γδ T-cell IFNγ production by statin initiation and BMI. **(a)** To assess whether BMI confounded differences in baseline IFNγ production between patients with T2D and non-diabetic controls (n=55), baseline IFNγ production was modeled with T2D status and baseline BMI as predictors. **(b)** To assess whether BMI confounded longitudinal treatment-associated changes in patients with T2D (n=25), change-score models included treatment group and baseline BMI as predictors, whereas baseline-adjusted models included baseline IFNγ production, treatment group, and baseline BMI. Treatment group was coded as SGLT2i alone versus SGLT2i plus GLP-1RA. Statin initiation was defined as absence of statin therapy at baseline followed by statin use at 12 months. β coefficients for categorical predictors represent adjusted differences in the percentage of IFNγ-producing cells, while β coefficients for continuous predictors represent the change in outcome per unit increase in the predictor. **(c)** In patients with T2D (n=25), the potential contribution of statin initiation during follow-up was assessed using change-score and baseline-adjusted models. Change-score models used the absolute change in IFNγ production from baseline to 12 months as the dependent variable and included treatment group and statin initiation as predictors. Baseline-adjusted models used IFNγ production at 12 months as the dependent variable and included baseline IFNγ production, treatment group, and statin initiation as predictors. BMI, body mass index; CI, confidence interval; GLP-1RA, glucagon-like peptide-1 receptor agonist; IFNγ, interferon-γ; SGLT2i, sodium-glucose cotransporter-2 inhibitor; T2D, type 2 diabetes.

Supplementary Table 6 Characteristics of patients at baseline and after 12 months of antidiabetic treatment. eGFR, estimated glomerular filtration rate; HDL, high-density lipoprotein; HOMA, homeostatic model assessment; LDL, Low-density lipoprotein.

Supplementary Figure 1 Flowchart of the study. GLP-1RA, glucagon-like peptide-1 receptor agonists; HbA_1c_, glycated hemoglobin A_1c_; mo, month; NA, not applicable; SGLT-2i, sodium-glucose co-transporter-2 inhibitors; T2DM, type 2 diabetes mellitus.

Supplementary Figure 2 Gating strategies. **(a)** Representative FACS plots of the gating strategy used to define δ_1_^+^ and δ_2_^+^ γδ T cells. **(b,c)** Representative FACS plots of **(b)** δ_1_^+^ and **(c)** δ_2_^+^ γδ T cells stimulated with Brefeldin A only or with PMA/Ionomycin and Brefeldin A.

Supplementary Figure 3 Phenotypic analysis of γδ T cells. **(a)** Frequency of CD3^+^δ_1_^+^ γδ T cells expressing CD27 or CD28, segregated by double-negative, single-positive and double-positive cells (n=25). **(b)** Frequency of CD3^+^δ_1_^+^ γδ T cells expressing CD16 (n=25). **(c)** Frequency of CD3^+^δ_1_^+^ γδ T cells expressing CD45RA (n=25). **(d)** Representative FACS plots of CD3^+^δ_1_^+^ cells from the same patient over time, including cells only stimulated with Brefeldin A (n=25). **(e)** Frequency of CD3^+^δ_2_^+^ γδ T cells expressing CD27 or CD28, segregated by double-negative, single-positive and double-positive cells (n=25). **(f)** Frequency of CD3^+^δ_2_^+^ γδ T cells expressing CD16 (n=25). **(g)** Frequency of CD3^+^δ_2_^+^ γδ T cells expressing CD45RA (n=25). **(h)** Representative FACS plots of CD3^+^δ_2_^+^ cells from the same patient over time, including cells only stimulated with Brefeldin A (n=25). **(i)** The cumulative amount of δ1^+^ and δ2^+^ γδ T cells as a percentage of CD3ϵ cells(n=25). **(j)** The ratio between δ1^+^ and δ2^+^ γδ T cells(n=25). An individual point represents one participant. Indicated are mean values ± standard error of mean and statistical significances at **p* < 0.050 and **<0.010 by one-way ANOVA with Tukey’s multiple comparisons post testing.

Supplementary Figure 4 Antidiabetic treatment does not impact TNF, IL-17A and Granzyme B production by γδ T cells. Groups were segregated based on treatment regimen and interferon (IFN) γ production was assessed using intracellular flow cytometry. **(a)** Tumor necrosis factor (TNF) and **(b)** interleukin (IL) 17A production by δ_1_^+^ γδ T cells at 0 and 12 months after antidiabetic treatment (n=15 and 10). **(c)** TNF and **(d)** Granzyme B production by δ_2_^+^ γδ T cells at 0 and 12 months after antidiabetic treatment (n=15 and 10).

Supplementary Figure 5 Reduced meta-inflammation is most prominent in patients with liver and kidney pathology at baseline. **(a)** *Post hoc* analysis of correlation between absolute levels of interferon (IFN) γ production and metabolic parameters, or relative change of these values (Δ) after 12 months of treatment (n=25). **(b–d)** *Post-hoc* analysis of the correlation between changes in interferon (IFN) γ production after 12 months of ant-diabetic treatment and the absolute levels of indicated parameters at baseline (prior to treatment) (n=25). **(b)** Heat-map of p values (n=25). Statistically significant changes are provided as numerical values. **(c-d)** Regression analysis correlating changes in IFNγ production with metabolic parameters for **(b)** δ_1_^+^ γδ T cells and **(c)** δ_2_^+^ γδ T cells (n=25). Regression analyses with statistically significant *p* value at <0.050 represented as mean and 95% confidence area of the best-fit line. ALT, alanine aminotransferase; AST, aspartate transaminase; BMI, body mass index; CRP, C-reactive protein; eGFR, estimated glomerular filtration rate FIB4, fibrosis-4 index; FPG, fasting plasma glucose; HbA_1c_, glycated hemoglobin A_1c_; HDL, high-density lipoprotein; HOMA-B, homeostatic model assessment beta cell function; HOMA-IR, homeostatic model assessment insulin resistance; HSI, hepatic steatosis index; LDL, low-density lipoprotein; NLFS, non-alcoholic fatty liver disease liver fat score; TyG, triglyceride index; WHR, waist-hip ratio.
