# Supplementary material for: γδ T-cell interferon-γ production reflects metabolic improvement during SGLT2 inhibitor-based therapy in type 2 diabetes
Source: Front Endocrinol (Lausanne). 2026 Sep 11;17:1922668. doi: 10.3389/fendo.2026.1922668 (PMC13612360; doi:10.3389/fendo.2026.1922668)
Supplement: Supplementary file 1 [file DataSheet1.pdf]

Table S1

Inclusion criteria	Exclusion criteria
1. Male and female gender	1. Patients with chronic inflammatory diseases (e.g. RA, IBD, COPD)
2. Age 18-79 years	2. Patients with latent autoimmune diabetes in adults
3. Clinical diagnosis of type 2 diabetes mellitus according to American Diabetes Association guidelines*	3. Patients taking anti-inflammatory or immunosuppressive drugs (e.g. corticosteroids)
4. HbA_{1c} ≥ 7.0% on current therapy	4. Patients with advanced liver disease (clinically evident signs of liver cirrhosis or LSM >12 kPa)
	5. Patients with chronic kidney disease of stages G3b** and worse, defined as estimated glomerular filtration rate <45 ml/min per $1.73m^2$
	6. Patients with acute infection/vaccinated in the past month
	7. Patients with C reactive protein >10 mg/l
	8. Patients with recurrent urinary tract infection
	9. Patients with active malignant disease
	10. Patients with diseases that impact the HPA-axis (e.g. Addison's disease, Cushing syndrome, etc.)
	11. Patients taking insulin or more than two oral antidiabetic drugs

*American Diabetes Association Professional Practice C. 2. Diagnosis and Classification of Diabetes: Standards of Care in Diabetes-2024. Diabetes care. 2024;47:S20-S42.
** KDIGO 2012 Clinical Practice Guideline for the Evaluation and Management of Chronic Kidney Disease. Kidney International Supplements. 2013;3(1):1–150. doi:10.1038/kisup.2012.73.

Table S2

	Included (n=25)	Excluded (n=24)	p value
Age, years	60±8 (44-74)	62±11 (23-73)	0.568
Female sex, n (%)	10 (40)	11 (46)	0.686
Glycated haemoglobin A_{1c}, %	8.3.±1.1	7.9.±1.2	0.234
Fasting plasma glucose, mmol/l	9.3±2.1	9.4±1.8	0.771
HOMA-insulin resistance	7.9±4.5	9.5±.4	0.470
Body mass index, kg/m^2	30.7±3.7	32.1±4.8	0.179
Body fat, %	29.8±8.7	33.0±8.3	0.195
C reactive protein, mg/l	2.9±2.4	3.3±2.0	0.563
Aspartate aminotransferase, U/l	29.2±13.5	34.1±19.1	0.297
Alanine aminotransferase, U/l	38.3±22.4	45.3±33.0	0.387
Vδ1 IFNγ cells (%)	54.8±16.8	50.7±20.3	0.447
Vδ2 IFNγ cells (%)	56.1±15.5	57.2±20.9	0.845

Table S3

Antibody (clone)	Source	Identifier
Anti-human CD3 monoclonal antibody Alexa Fluor 700 (OKT3)	eBioScience, Inc., San Diego, California, United States	56-0037-42
Fixable Viability Dye eFluor 506	eBioScience, Inc., San Diego, California, United States	65-0866-14
Anti-human CD45RA monoclonal antibody SB645 (HI100)	eBioScience, Inc., San Diego, California, United States	64-0458-42
Anti-human CD27 monoclonal antibody FITC (LG.7F9)	eBioScience, Inc., San Diego, California, United States	11-0271-82
Anti-human CD28 monoclonal antibody PE (CD28.2)	eBioScience, Inc., San Diego, California, United States	12-0289-42
Anti-human IFN-γ monoclonal antibody FITC (4S.B3)	eBioScience, Inc., San Diego, California, United States	11-7319-82
Anti-human TNF-α monoclonal antibody PE-Cyanine7 (MAb11)	eBioScience, Inc., San Diego, California, United States	25-7349-82
Anti-human Granzyme B monoclonal antibody PE (N4TL33)	eBioScience, Inc., San Diego, California, United States	12-8896-42
Anti-human Granzyme B monoclonal antibody eFluor 450 (N4TL33)	eBioScience, Inc., San Diego, California, United States	48-8896-42
Anti-human CD16 monoclonal antibody APC-eFluor 780 (CB16)	eBioScience, Inc., San Diego, California, United States	47-0168-42
Anti-human CD337 monoclonal antibody eFluor 450 (AF29-4D12)	eBioScience, Inc., San Diego, California, United States	48-3379-42
Anti-human CD314 monoclonal antibody PerCP-eFluor 710 (1D11)	eBioScience, Inc., San Diego, California, United States	46-5878-42
Anti-human CD69 monoclonal antibody SB780 (FN50)	eBioScience, Inc., San Diego, California, United States	78-0699-42
Anti-human TCRα/β monoclonal antibody PE-eFluor 610 (IP26)	eBioScience, Inc., San Diego, California, United States	61-9986-42
Anti-human IL-17A monoclonal antibody APC (eBio64DEC17)	eBioScience, Inc., San Diego, California, United States	17-7179-42
Anti-human TCR Vδ1 monoclonal antibody PerCP-Vio 770 (REA173)	Miltenyi Biotec, Bergisch Gladbach, Germany	130-120-440
Anti-human TCR Vδ2 monoclonal antibody PE (REA771)	Miltenyi Biotec, Bergisch Gladbach, Germany	130-111-010
Anti-human CD197 monoclonal antibody Brilliant Violet 421 (G043H7)	BioLegend, San Diego, California, United States	353208

Clones indicated in parentheses. APC, allophycocyanin; FITC, fluorescein isothiocyanate; IFN, interferon; IL, interleukin; PE, phycoerythrin; PerCP, peridinin chlorophyll-A protein; SB, super bright; TCR, T cell receptor; TNF, tumor necrosis factor.

Table S4

	Control (n=30)	Diabetes (n=25)	p value
Age, years	58±13 (53–68)	60±8 (44-74)	0.362
Female sex, n (%)	12 (40)	10 (40)	0.999
Glycated haemoglobin A_{1c}, %	5.4±0.4	8.3.±1.1	
Fasting plasma glucose, mmol/l	5.6±0.6	9.3±2.1	<0.001
HOMA-insulin resistance	3.00±1.88	7.9±4.5	<0.001
HOMA-beta cell function	11.65±5.26	7.52±4.1	0.003
Quantitative insulin sensitivity check index	0.34±0.03	0.29±0.02	<0.001
Body mass index, kg/m^2	27.1±4.0	30.7±3.7	0.001
Body fat, %	28.1±8.4	29.8±8.7	0.093
Skeletal muscle index, kg/m^2	8.0±1.0	8.8±1.3	0.014
Waist-hip ratio	0.90±0.09	0.97±0.07	0.021
Leukocyte count, $\times 10^9$ per litre	6.4±1.7	7. 7±2.2	0.013
C reactive protein, mg/l	2.5±2.5	2.9±2.4	0.542
Triglycerides, mmol/l	1.2±0.6	1.8±1.2	0.021
HDL cholesterol, mmol/l	1.6±0.5	1.2±0.4	0.001
LDL cholesterol, mmol/l	3.1±1.2	2.8±1.0	0.299
Total cholesterol, mmol/l	5.1±1.5	4.6±1.3	0.212
Statin use, n (%)	10 (33.33)	13 (52)	0.168
Aspartate aminotransferase, U/l	26.6±6.0	29.2±13.5	<0.001
Alanine aminotransferase, U/l	27.4±7.3	38.2±22.4	0.015
Hepatic steatosis index	38.1±5.1	44.1±6.1	0.008
Triglyceride-glucose index	4.59±0.25	9.3±0.6	<0.001
NAFLD-liver fat score	1.1±1.0	2.5±1.8	0.002

Table S5

a

γδ T cell subset	Predictor	β	95% CI	P value
Vδ1⁺	GLP-1RA add-on	**12.82**	**3.23 to 22.41**	**0.01**
	Statin initiation	−0.52	−1.65 to 0.61	0.36
Baseline-adjusted model – Vδ1⁺	Baseline IFNγ	3.94	−6.72 to 14.60	0.461
	GLP-1RA add-on	−0.57	−1.82 to 0.69	0.37

b

Model – γδ T cell subset	Predictor	β	95% CI	P value
Change-score model – Vδ1⁺	GLP-1RA add-on	8.88	−8.18 to 25.94	0.292
	Baseline BMI	−0.52	−2.82 to 1.78	0.644
Baseline-adjusted model – Vδ1⁺	Baseline IFNγ	0.842	0.326 to 1.358	0.003
	GLP-1RA add-on	8.19	−9.30 to 25.68	0.341
	Baseline BMI	−0.81	−3.32 to 1.71	0.513
Change-score model – Vδ2⁺	GLP-1RA add-on	3.15	−13.68 to 19.98	0.702
	Baseline BMI	0.02	−2.25 to 2.30	0.982
Baseline-adjusted model – Vδ2⁺	Baseline IFNγ	0.491	−0.012 to 0.993	0.055
	GLP-1RA add-on	3.17	−12.52 to 18.86	0.679
	Baseline BMI	−1.04	−3.40 to 1.33	0.371

c

Model – γδ T cell subset	Predictor	β	95% CI	P value
Change-score model – Vδ1⁺	GLP-1RA add-on	6.11	−8.89 to 21.11	0.408
	Statin initiation	−7.20	−23.57 to 9.16	0.371
Baseline-adjusted model – Vδ1⁺	Baseline IFNγ	0.941	0.455 to 1.427	**<0.001**
	GLP-1RA add-on	5.53	−10.56 to 21.62	0.483
	Statin initiation	−6.79	−23.90 to 10.31	0.418
Change-score model – Vδ2⁺	GLP-1RA add-on	1.13	−12.44 to 14.69	0.865
	Statin initiation	−15.82	−30.62 to −1.02	**0.037**
Baseline-adjusted model – Vδ2⁺	Baseline IFNγ	0.764	0.256 to 1.272	**0.005**
	GLP-1RA add-on	−0.04	−13.90 to 13.81	0.995
	Statin initiation	−11.77	−29.00 to 5.46	0.17

Table S6

	Baseline			12 Months				
	**SGLT2i (*n*=15)**	**SGLT2i/ GLP1ra (*n*=10)**	***p* value**	**SGLT2i (*n*=15)**	**SGLT2i/ GLP1ra (*n*=10)**	***p* value**	**SGLT2i 0 vs. 12 months p value**	**SGLT2i /GLP1Ra 0 vs. 12 months p value**
Age, Years	63.7±7 (48-74)	53.4±15 (44-68)						
Female sex, n (%)	6 (40)	4 (40)						
Glycated haemoglobin A_{1c}, %	8.1±1.0	7.9±2.2	0.252	6.7±0.3	6.0±1.7	0.055	**>0.001**	**>0.001**
Fasting plasma glucose, mmol/l	8.6±1.2	9.5±3.4	**0.038**	7.0±0.9	6.5±1.8	0.419	**>0.001**	**0.007**
HOMA-insulin resistance	7.9±5.1	8.3±3.3	0.975	4.2±3.2	5.3±3.6	0.631	**0.016**	**0.003**
HOMA-beta cell function	8.1±3.6	6.6±4.1	0.424	7.2±4.0	9.1±5.0	0.235	0.356	**0.028**
Quantitative insulin sensitivity check index	0.29±0.02	0.27±0.07	0.526	0.33±0.04	0.29±0.08	0.633	**0.001**	**0.003**
Body mass index, kg/m^2	29.3±8.4	30.2±8.4	**0.017**	27.5±3.2	27.6±7.4	0.091	**>0.001**	**0.009**
Body fat, %	28.5±7.9	29.6±10.3	0.355	28.9±8.7	29.1±9.0	0.698	0.711	0.520
Skeletal muscle index, kg/m^2	8.8±1.4	8.2±2.3	0.993	8.0±1.3	7.9±2.0	0.446	**>0.001**	0.275
Waist-hip ratio	0.96±0.06	0.91±0.27	0.517	0.96±0.05	0.91±0.25	0.806	0.957	0.448
Leukocyte count, $\times 10^9$ per litre	7.6±1.6	7.5±3.0	0.672	7.9±1.6	8.3±3.0	0.263	0.311	0.113
C reactive protein, mg/l	2.3±1.8	3.6±2.6	0.111	1.9±2.1	1.9±1.4	0.957	0.648	0.083
Triglycerides, mmol/l	1.8±1.3	2.1±1.3	0.995	1.6±1.0	1.8±1.0	0.999	0.220	0.347
HDL cholesterol, mmol/l	1.2±0.4	1.2±0.3	0.946	1.4±0.3	1.3±0.4	0.934	**0.012**	**0.012**
LDL cholesterol, mmol/l	2.7±1.0	2.8±0.9	0.527	2.1±1.0	2.1±1.2	0.822	**0.022**	**0.074**
Total cholesterol, mmol/l	4.5±1.4	4.6±1.4	0.636	8.1±1.0	8.1±1.0	0.911	0.128	0.115
Statin use, *n* (%)	9 (60)	7 (70)	0.680	14 (93)	9 (90)	1.000	0.063	0.500
Aspartate aminotransferase, U/l	29.7±14.5	30.6±15.3	0.823	27.3±9.9	25.3±11.6	0.577	0.459	0.399
Alanine aminotransferase, U/l	36.7±22.6	42.0±21.8	0.674	29.7±11.2	26.6±13.0	0.306	0.131	0.060
Hepatic steatosis index	41.6±4.6	43.6±12.6	**0.011**	39.2±4.3	38.2±10.8	0.360	**0.008**	**>0.001**
Triglyceride-glucose index	9.2±0.7	8.9±2.4	0.262	8.9±0.6	8.4±2.3	0.916	**0.027**	**0.020**
NAFLD-liver fat score	2.6±1.9	2.6±1.6	0.795	1.3±1.5	1.8±1.7	0.745	**0.014**	**0.049**
Urea, mmol/l	6.3±1.3	5.2±1.5	0.100	7.1±1.8	5.6±2.0	0.066	0.114	0.613
Creatinine, µmol/l	77.5±10.6	70.1±22.6	0.546	79.2±15.9	67.5±23.4	0.158	0.573	**0.030**
eGFR, ml/min/1.73m^2	78.6±12.2	83.1±25.8	0.063	81.5±13.6	86.0±28.0	0.054	0.379	**0.046**

Figure S1

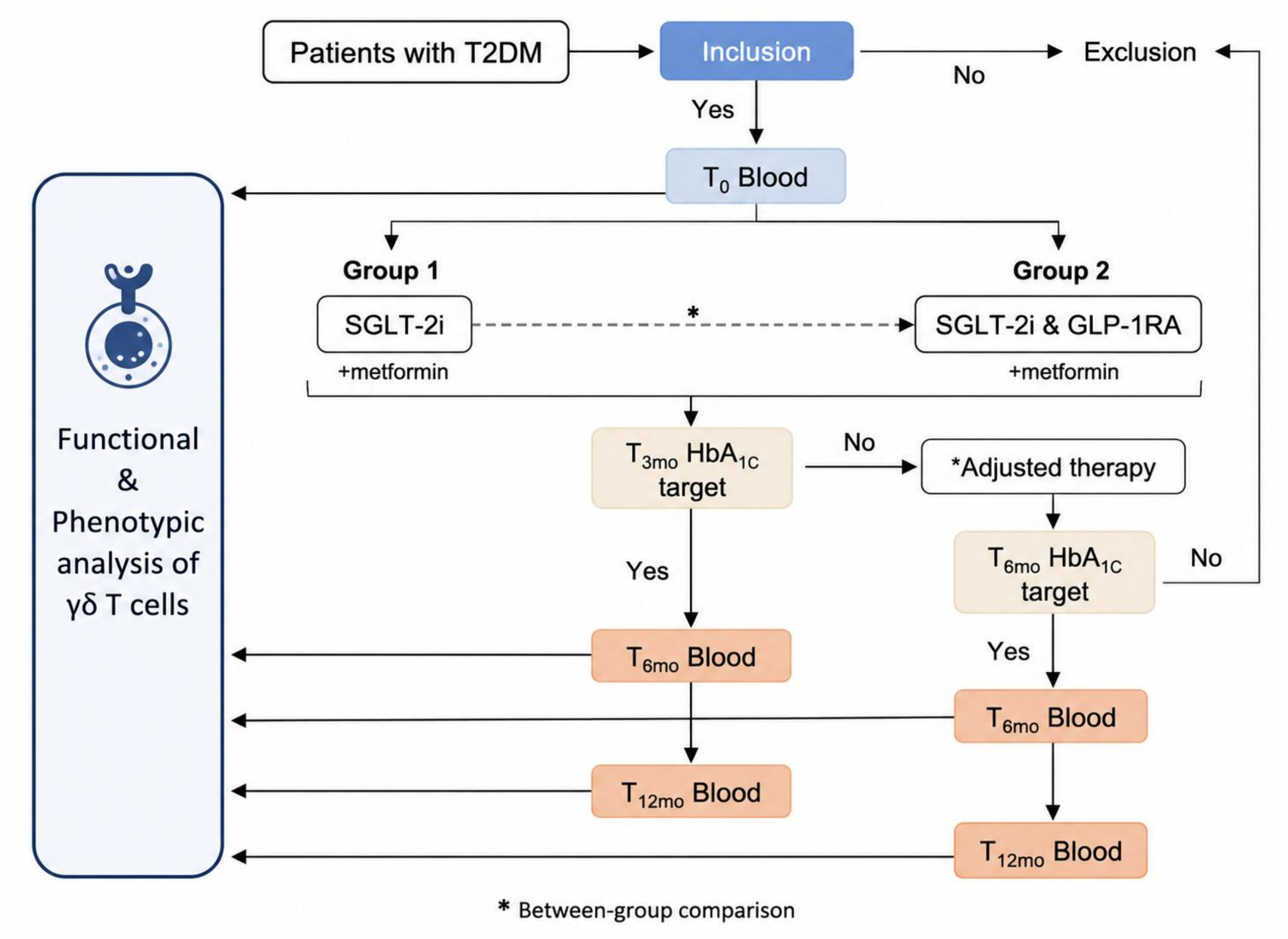

Patients with T2DM
Inclusion
Yes
No
Exclusion
T0 Blood
Group 1
SGLT-2i
+metformin
*
Group 2
SGLT-2i & GLP-1RA
+metformin
T3mo HbA1C target
No
*Adjusted therapy
Yes
T6mo HbA1C target
No
Yes
T6mo Blood
T6mo Blood
T12mo Blood
T12mo Blood
Functional & Phenotypic analysis of γδ T cells
* Between-group comparison

Figure S2

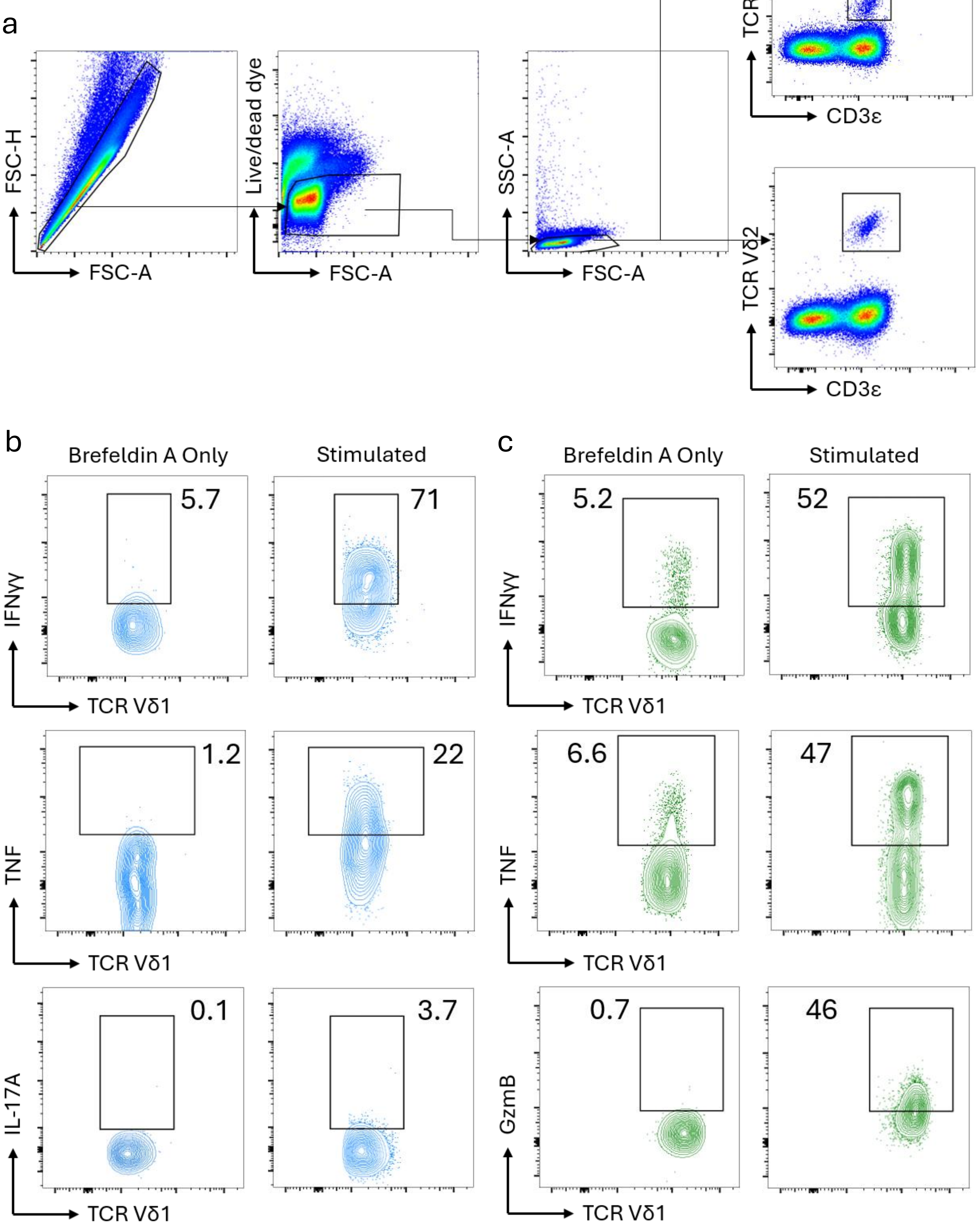
a
FSC-H
FSC-A
Live/dead dye
FSC-A
SSC-A
FSC-A
TCR Vδ1
CD3ε
TCR Vδ2
CD3ε
b
Brefeldin A Only
Stimulated
5.7
71
IFNγ
TCR Vδ1
1.2
22
TNF
TCR Vδ1
0.1
3.7
IL-17A
TCR Vδ1
c
Brefeldin A Only
Stimulated
5.2
52
IFNγ
TCR Vδ1
6.6
47
TNF
TCR Vδ1
0.7
46
GzmB
TCR Vδ1

Figure S3

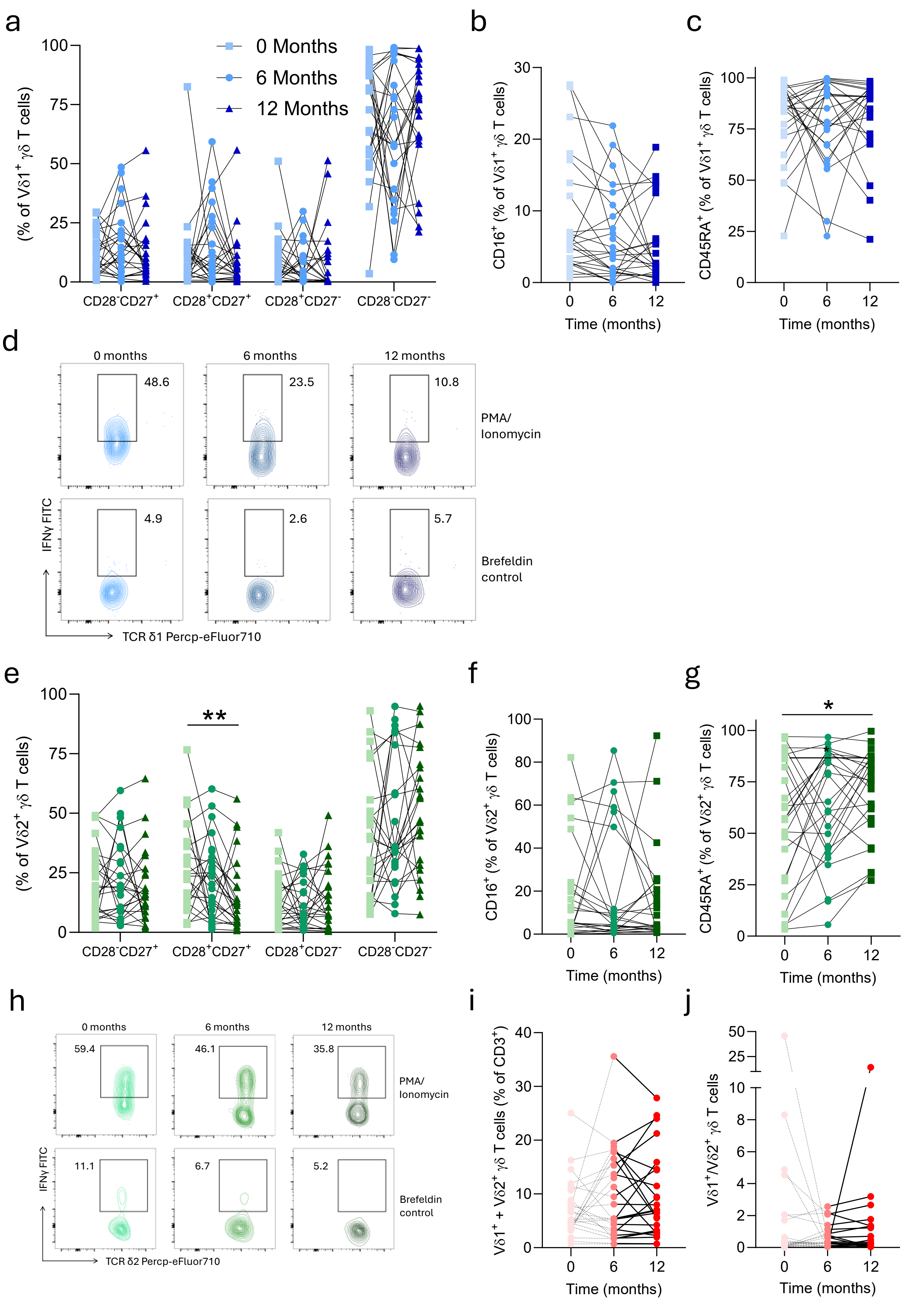

a
0 Months
6 Months
12 Months
(% of Vδ1+ γδ T cells)
CD28-CD27+
CD28+CD27+
CD28+CD27-
CD28-CD27-
b
CD16+ (% of Vδ1+ γδ T cells)
Time (months)
c
CD45RA+ (% of Vδ1+ γδ T cells)
Time (months)
d
0 months
6 months
12 months
48.6
23.5
10.8
PMA/
Ionomycin
4.9
2.6
5.7
Brefeldin
control
IFNγ FITC
TCR δ1 Percp-eFluor710
e
(% of Vδ2+ γδ T cells)
**
CD28-CD27+
CD28+CD27+
CD28+CD27-
CD28-CD27-
f
CD16+ (% of Vδ2+ γδ T cells)
Time (months)
g
CD45RA+ (% of Vδ2+ γδ T cells)
*
Time (months)
h
0 months
6 months
12 months
59.4
46.1
35.8
PMA/
Ionomycin
11.1
6.7
5.2
Brefeldin
control
IFNγ FITC
TCR δ2 Percp-eFluor710
i
Vδ1+ + Vδ2+ γδ T cells (% of CD3+)
Time (months)
j
Vδ1+/Vδ2+ γδ T cells
Time (months)

Figure S4

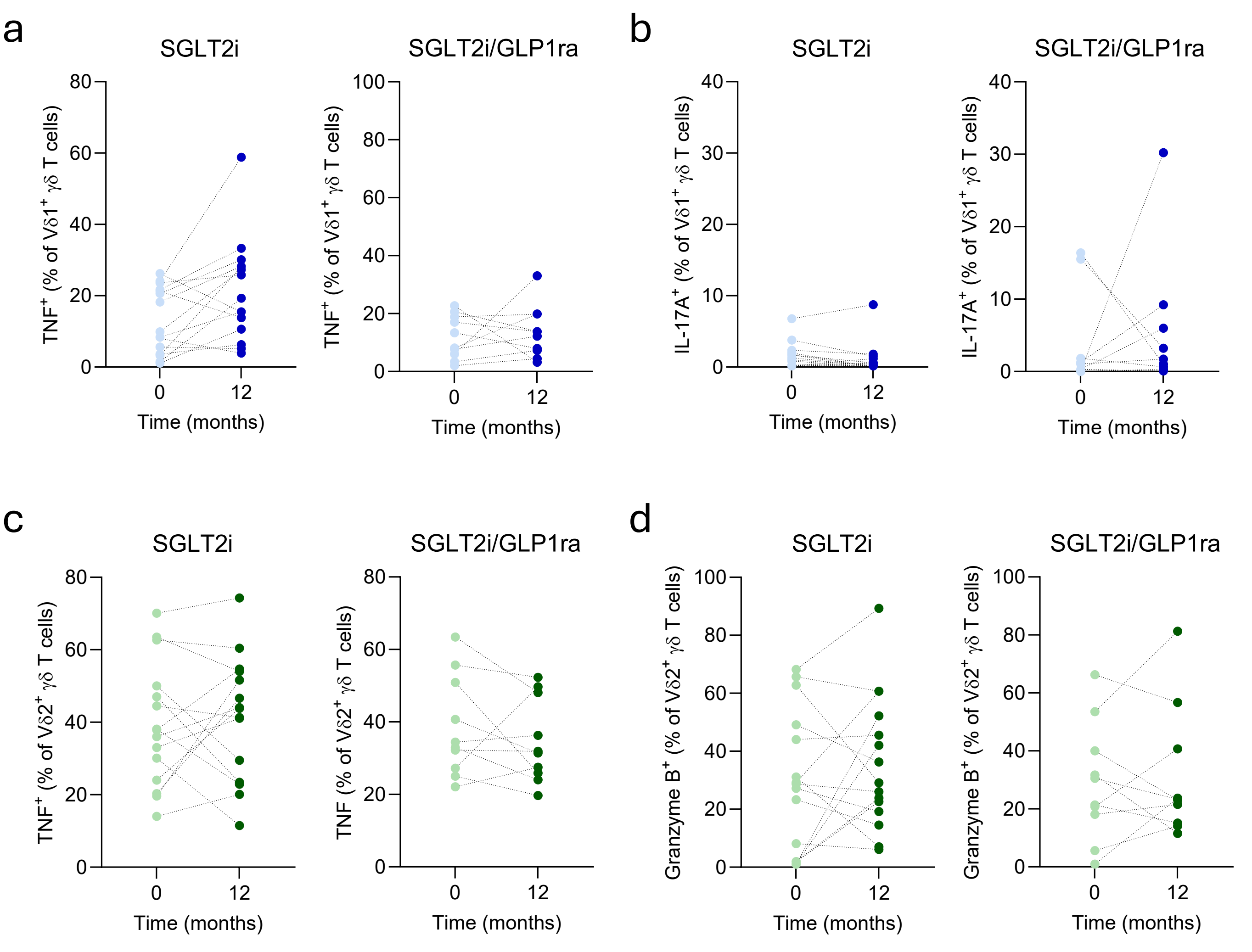
a
SGLT2i
TNF$^+$ (% of Vδ1$^+$ $\gamma\delta$ T cells)
Time (months)
SGLT2i/GLP1ra
b
SGLT2i
IL-17A$^+$ (% of Vδ1$^+$ $\gamma\delta$ T cells)
SGLT2i/GLP1ra
c
SGLT2i
TNF$^+$ (% of Vδ2$^+$ $\gamma\delta$ T cells)
SGLT2i/GLP1ra
TNF (% of Vδ2$^+$ $\gamma\delta$ T cells)
d
SGLT2i
Granzyme B$^+$ (% of Vδ2$^+$ $\gamma\delta$ T cells)
SGLT2i/GLP1ra

Figure S5

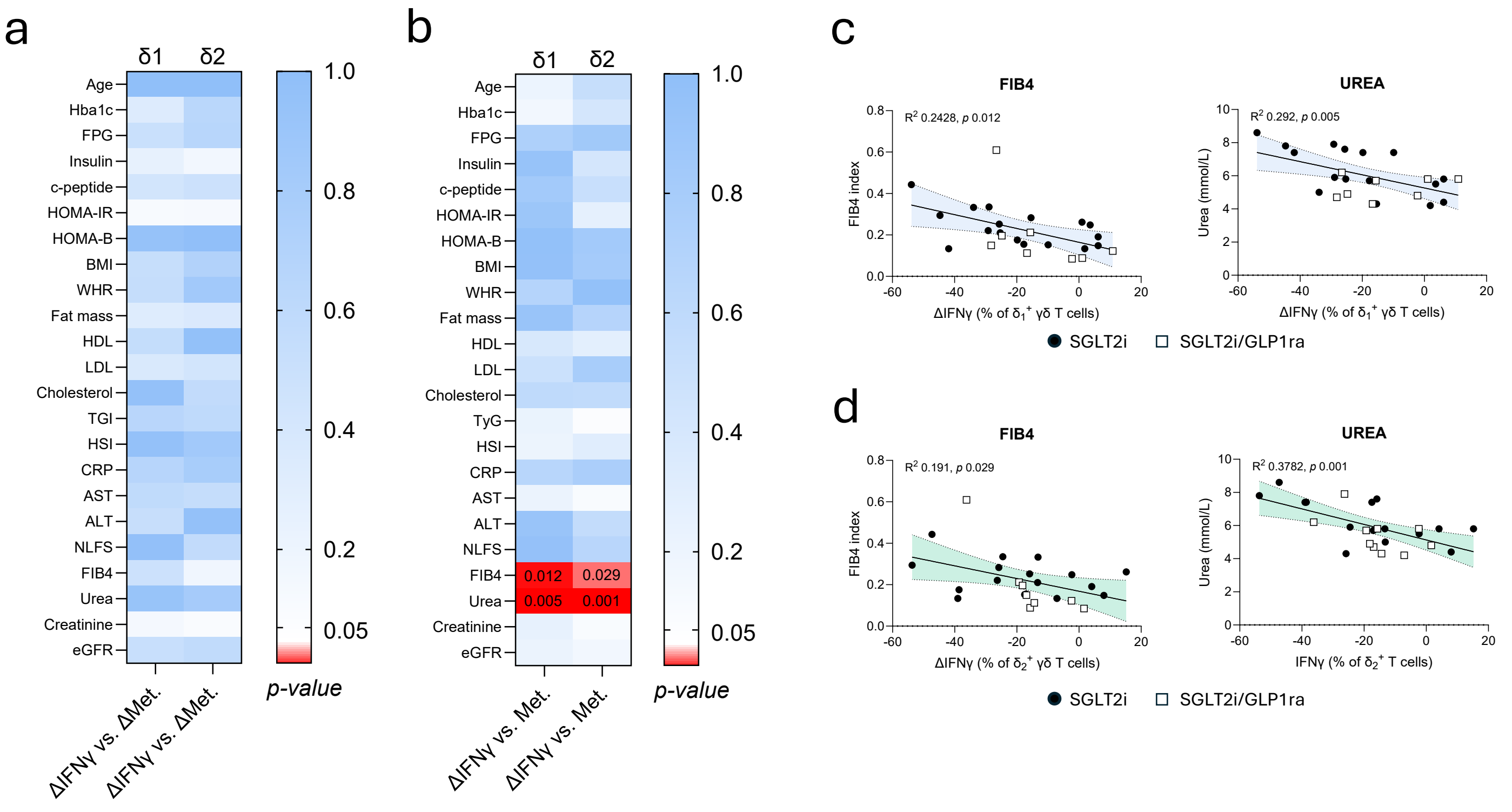

a
δ1
δ2
Age
Hba1c
FPG
Insulin
c-peptide
HOMA-IR
HOMA-B
BMI
WHR
Fat mass
HDL
LDL
Cholesterol
TGI
HSI
CRP
AST
ALT
NLFS
FIB4
Urea
Creatinine
eGFR
ΔIFNγ vs. ΔMet.
ΔIFNγ vs. ΔMet.
1.0
0.8
0.6
0.4
0.2
0.05
p-value
b
δ1
δ2
Age
Hba1c
FPG
Insulin
c-peptide
HOMA-IR
HOMA-B
BMI
WHR
Fat mass
HDL
LDL
Cholesterol
TyG
HSI
CRP
AST
ALT
NLFS
FIB4
Urea
Creatinine
eGFR
0.012
0.029
0.005
0.001
ΔIFNγ vs. Met.
ΔIFNγ vs. Met.
1.0
0.8
0.6
0.4
0.2
0.05
p-value
c
FIB4
R² 0.2428, p 0.012
FIB4 index
ΔIFNγ (% of δ1⁺ γδ T cells)
UREA
R² 0.292, p 0.005
Urea (mmol/L)
ΔIFNγ (% of δ1⁺ γδ T cells)
SGLT2i
SGLT2i/GLP1ra
d
FIB4
R² 0.191, p 0.029
FIB4 index
ΔIFNγ (% of δ2⁺ γδ T cells)
UREA
R² 0.3782, p 0.001
Urea (mmol/L)
IFNγ (% of δ2⁺ T cells)
SGLT2i
SGLT2i/GLP1ra
